# Deciphering the cardiac neuron landscape in heart failure patients

**DOI:** 10.1371/journal.pcbi.1014082

**Published:** 2026-03-20

**Authors:** Shuping Zhuang, Xiuqi Yang, Nan Zhang, JiangQi Liu, Kaidong Liu, Huiming Han, Songmei Zhai, Mingyue Liu, Haihai Liang, Yunyan Gu, Yanjie Lu

**Affiliations:** 1 State Key Laboratory of Frigid Zone Cardiovascular Diseases (SKLFZCD), Department of Pharmacology, College of Pharmacy, Harbin Medical University, Harbin, Heilongjiang, China; 2 State Key Laboratory of Frigid Zone Cardiovascular Diseases (SKLFZCD), Department of Systems Biology, College of Bioinformatics Science and Technology, Harbin Medical University, Harbin, Heilongjiang, China; Nanjing University, CHINA

## Abstract

Neurons exert a pivotal role in the preservation of cardiac physiological function. However, there is a lack of explanation about the mechanism of cardiac neurons in the pathogenesis of cardiac dysfunction. Here, we generated a cardiac neuron landscape including 11,026 neuronal cells based on the integration of published single-nucleus RNA sequencing data from 75 patients with heart failure and 45 healthy donors. We determined ten distinct neuronal cell subsets differing in abundances, compositions, and biological functions in the heart. In particular, N4-ALK neurons were significantly enriched in failing hearts relative to healthy controls, and their abundance was associated with the response to left ventricular assist device implantation. *RXRG*, a transcription factor highly expressed in neuronal cells, participated in the transcriptional regulatory network of N4-ALK neurons and showed a positive correlation with the expression of their marker genes. Notably, in heart failure, the PTN-PTPRZ1 axis mediated specific crosstalk between cardiac fibroblasts and N4-ALK neurons. Finally, we used N4-ALK-related features to develop an optimized prediction model for identifying individuals with heart failure. Overall, our integrative cardiac neuron atlas comprehensively characterizes the molecular and functional diversity of neuronal cells, providing a new perspective for further exploration of the regulatory function of neurons in heart failure.

## Author summary

The heart is a highly innervated organ and is innervated by both sympathetic and parasympathetic nerves, which is crucial for cardiac contraction and electrical signal transduction. Single-cell RNA sequencing offers a powerful resolution for dissecting cardiac neuromodulation by enabling the high-resolution characterization of specific neuronal populations. However, a generalized landscape of neurons in heart failure still remains insufficient. Here, our study systematically integrated current resources of neurons across heart failure researches, providing a perspective on the heterogeneity of cardiac neurons. Our findings first identified a subset of neurons, named N4-ALK, as a heart failure-associated neuronal subset, elucidated their disease-promoting role via the RXRG-PTN-PTPRZ1 axis, and offered new insights into heart failure pathophysiology. Beyond dissecting the underlying molecular mechanisms, we developed an N4-ALK-derived signature within a machine-learning framework to identify heart failure patients at the population level.

## Introduction

Heart failure is a clinical syndrome in most patients with end-stage heart disease, mainly consisting of dilated cardiomyopathy (DCM) and hypertrophic cardiomyopathy (HCM) [1]. Patients with heart failure typically present with a decline in cardiac pumping function, difficulty breathing, and physical weakness. As reported, there has been an increased number of patients diagnosed with heart failure [2]. Moreover, patients suffering from heart failure harbor a 5-year survival rate around 50% after diagnosis [3]. During the pathological process of the heart, typical characteristics such as cardiomyocyte hypertrophy, fibroblast activation, and extracellular matrix deposition often appear to cope with cardiac injury [4,5]. Except for that, some cells, such as neurons involved in sympathetic nervous system, with low abundance in heart microenvironment are always overlooked.

The sustained activation of the sympathetic nervous system leads to chronic contraction of the heart and an increase in blood volume, ultimately accelerating the process of heart damage and dysfunction [6,7]. Tampakakis *et al.* found inhibition of sympathetic innervation increased heart size and cardiomyocyte quantity, which makes sense to develop neuromodulation strategies toward cardiac regeneration [8]. A previous study has identified astroglia-like cardiac nexus glia, marked by GFAP, with an essential role in regulating heart rate and rhythm in species including zebrafish, mouse, and human beings [9]. Thus, linking neuronal cells or glia-like cells with nerve innervation of the heart is meaningful for understanding the pathogenesis of heart failure at the cellular level. In addition, considering that patients with advanced-stage heart failure often suffer from severe cardiac fibrosis, exploring the relationship between abnormal neural signals and cardiac fibrosis seems meaningful [10]. Notably, Mias *et al.* demonstrated that myocardial infarction-derived myofibroblasts exhibit higher expression level of nerve growth factor and also promote PC12 cells differentiation, underscoring the essential role of fibroblasts in the stability of sympathetic nervous system innervation [11].

Given that single-cell transcriptome data have provided the possibility to explore cell composition and associated gene expression and in-depth analysis of highly heterogeneous of heart tissue. In 2020, a large-scale cellular landscape of adults heart noted that the number of neurons that make up the components of the cardiac microenvironment accounts for about 1% and these cells are marked by *PLP1*, *NRXN1*, and *NRXN3* [12]. Besides, the work of Koenig *et al.,* which contains sequencing data from heart failure samples, also showed neurons occupy 0.8% of the whole heart tissue [13]. In recent years, researchers have paid more attention to the effects of the activation and proliferation of cardiac fibroblasts on the course of heart disease [14,15]. Cell types such as cardiomyocytes, endothelial cells, and macrophages, which make up a large proportion of the heart tissue, have also been widely studied for their roles in regulating heart damage [16–18]. In contrast, limited research focuses on the characterization of neurons in response to cardiac injury based on high-throughput data. Recently, Cui *et al.* firstly explored the heterogeneity of neurons in heart failure with DCM [19]. However, in that study, the quantity of neurons was still insufficient to completely characterize cellular features of cardiac neurons, especially the features that differ from normal hearts. In summary, a generalized landscape of neurons in heart failure still remains deficient.

In this study, based on the integration of single-nucleus RNA sequencing (snRNA-seq) datasets from patients with heart failure, mostly including DCM and HCM samples, we obtained a total of 11,026 neuronal cells. We explored intracellular heterogeneity in neurons through analysis of their differentiation relationships, biological functions, and different transcriptional regulation networks. Besides, we proposed that interactions between cardiac fibroblasts and heart failure-associated neuronal cells play a significant role in contributing to heart disease. Lastly, based on machine learning algorithms, we developed a model to identify individuals with heart failure at the transcriptome level.

## Materials and methods

### Data processing of human heart snRNA-seq datasets

All human heart tissue snRNA-seq datasets used in this study were summarized in S1 Table, including their designated names for subsequent analysis, sample types, and data sources.

The preprocessing workflow for snRNA-seq datasets was as follows. We constructed an object by using Seurat (version 4.4.0) package [20]. Cells expressing fewer than 200 genes and genes detected in less than three cells were excluded. After quality control, we performed normalization of the count matrix, selection of 2,000 variable features, data standardization and dimensionality reduction using principal-component analysis on the integrated Seurat object. Harmony (version 1.0.3) [21] algorithm was used to remove batch effects across different datasets or conditions. The number of principal components for the integration was determined based on the Elbow Plot inflection point, and all remaining parameters were maintained at their default settings. After that, we used FindClusters function to identify clusters and UMAP algorithm for visualizing the reduction result. We performed cell type annotation according to the canonical markers [13]. Notably, for data integration, commonly detected genes were used to construct the integrated object.

### Construction and exploration of the neuronal atlas

To construct a neuronal atlas, we firstly integrated three data cohorts from the works of Koenig *et al.* [13], Liu *et al.* [22] as well as Reichart *et al.* [23] (S1 Table). In the subsequent analysis, these three sets were ultimately labeled as GSE183852, Liu *et al.*, and Reichart *et al.*, respectively. Utilizing principal components 1–27, we applied the Harmony algorithm to perform batch correction on different datasets. After cell type annotations, we extracted cells labeled as “Neuronal” to perform unsupervised clustering. Then, we used clustree package (version 0.5.0) to seek an optimum classification resolution in performing neurons clustering. We set the resolution from 0.1 to 1 with an interval of 0.1 and finally chose 0.1 for determining clusters. After that, we employed the function FindAllMarkers to identify differential expression genes in each cluster, with thresholds defined as log-fold change (logFC) > 0.25 and Benjamini-Hochberg (BH)-adjusted *P*-value < 0.05. Gene Ontology (GO) enrichment analysis was performed to reveal the different functions among these subsets by using clusterProfiler package (version 4.10.1). We considered a BH-adjusted *P*-value of less than 0.05 as indicating significant enrichment.

### Condition distribution preference for different cell types

We calculated the ratio of observed over expected cell numbers (Ro/e) for each cell type in different conditions, including heart failure (DCM and HCM) and healthy samples. A Ro/e value greater than one indicates enrichment of this cell type under the given condition.

### Validation of the neuronal subsets in SCP1303 cohort

To verify the reliability of previously identified neuronal subsets, we obtained another snRNA-seq dataset SCP1303 conducted by Chaffin *et al.* to reproduce the molecular subtyping. We set the resolution from 0.1 to 0.5 with an interval of 0.05 and finally chose 0.2 to perform unsupervised clustering on neuronal cells. After identifying distinct clusters and cluster-specific differentially expressed genes, we calculated the number of overlapping genes between SCP1303 clusters and neuronal subsets to assess their molecular similarities. Hypergeometric test was used to test the significance of overlapping genes with *P* value less than 0.05. For further confirming their similarities, we employed the AddModuleScore function included in Seurat package to evaluate the average expression of marker genes from N1-XKR4, N2-OGFRL1, and N4-ALK in SCP1303 neuronal cells.

### Pseudo time trajectory analysis

We used Monocle2 (version 2.28.0) to infer pseudo time trajectory of neuronal cells. Firstly, the UMI matrix was used as input, and the trajectory was constructed using the differential expression genes from each subset. Secondly, we used the BEAM function to identify genes that significantly change their expressions along the trajectory determined by the branch site (*q* < 1 × 10^−4^). Last, GO enrichment analyses of differential expression genes at different branch sites were performed using clusterProfiler package (version 4.10.1) by controlling the adjusted *P*-value less than 0.05. Notably, we used CytoTRACE and Slingshot algorithms to complement Monocle2’s analysis of neuronal lineage differentiation.

### Cell label transferring analysis

We performed a clinical association analysis of neuronal subsets using data from GSE226314, with details provided in S1 Table. Firstly, batch effects between different samples were removed using Harmony. Next, we used the functions FindTransferAnchors and TransferData in Seurat to annotate the label of neuronal cells in GSE226314 with the integrated neuronal atlas as reference data. Wilcoxon rank-sum test was used to compare the differential expression of marker genes from N4-ALK between left ventricular assist device (LVAD) responders and non-responders.

We collected another three human heart snRNA-seq datasets, SCP1849, Linna-Kuosmanen *et al.*, and Kuppe *et al.* (S1 Table), containing several heart disease events for analysis and validation. Following the preprocessing workflow described above, we generated new neuronal data. Then, the integrated neuronal atlas was also used as a reference to annotate these newly added neuronal cells into established subsets using functions of FindTransferAnchors and TransferData in Seurat. We used factoextra (version 1.2.3) package to perform comparisons of neuronal cell subsets in different datasets after cell label transfer. Meanwhile, we chose Pearson correlation analysis and ‘ward.D2’ method to complete the above analysis. Wilcoxon rank-sum test was used to compare the differential expression of N4-ALK associated molecules (*PTPRZ1*, *ALK*, *LRRC4C*, and *RXRG*) between failing and healthy hearts.

### Cell-cell communication analysis

We used CellChat package (version 1.6.1) to analyze cellular interactions between different cell types. We obtained ligand-receptor pair relationships from CellChatDB.human database. The neuronal subset with fewer than ten cells was excluded in the following analysis. Utilizing data from 45 healthy controls and 75 heart failure patients, we constructed three CellChat objects derived from DCM, HCM, and normal groups, respectively. And the three CellChat objects were then merged into one for subsequent comparisons. We calculated the network centrality score of each cell type in all signaling pathways by using the function computeCommunProbPathway. The differences in interaction strength among cell types were compared between DCM and healthy groups as well as HCM and healthy groups in the integrated atlas. Whereas, in the cell label transferring analysis, we focused on the differential interactions of PTN signaling among cell types between newly added heart failure group and healthy group.

Cross-validation of CellChat predictions was performed using the NicheNet R package (version 2.2.1.1). The necessary prior knowledge networks, including ligand-receptor interactions, ligand-target gene regulatory potential scores, and weighted_networks, was downloaded from Zenodo (https://doi.org/10.5281/zenodo.3260758).

### Spatial transcriptomics analysis

Spatial transcriptome data were also obtained from Liu *et al.* [22], comprising samples from HCM patients. To determine the expression status of a gene of interest for each spot, we classified it as “high” or “low” based on the median expression value. Spots with expression levels higher than the median were designated as “high” and the others as “low”. For validating co-expression of gene pairs, we defined four expression statuses for each spot, including “high-high” (high expression of both genes at the same spot), “low-low” (low expression of both genes at the same spot), and “high-low” or “low-high” (discordant high or low expression of the two genes).

### pySCENIC regulon analysis

We employed pySCENIC to identify key transcription factors in regulating the function of neuronal subsets. Apart from setting the seed 666, we ran the standard process of pySCENIC with default parameters to acquire heterogeneous regulatory networks based on the integrated neuronal map. Then, the regulon specificity score was calculated by the calcRSS function from SCENIC package (version 1.3.1). The top five transcription factors with higher regulon specificity score in each subset were used for subsequent analysis.

### Developing a predictive model for heart failure via machine learning

According to the Leave-One-Out Cross-Validation (LOOCV) framework, used in training classification models [24], to construct a model for the identification of heart failure population. In this study, nine machine learning methods for integration included Least absolute shrinkage and selection operator (Lasso), Ridge, Elastic Net (Enet), Support Vector Machine (SVM), Linear Discriminant Analysis (LDA), Random Forest (RF), Classification and Regression Tree (CART), Quadratic Discriminant Analysis (QDA), and Naive Bayes (NB). Following Zhu *et al.* [24], 40 integration models were constructed under the LOOCV framework. These models comprised 17 single-algorithm strategies (including Enet with different alpha thresholds) based on the aforementioned algorithms, 16 RF-based models, and seven LASSO-based models. RF and Lasso algorithms, utilizing default parameter settings, were employed for feature selection. For RF-based models, only features with an importance score greater than one were retained. For LASSO-based models, only features with non-zero lambda coefficients were selected. We utilized four independent transcriptomic datasets (GSE141910, GSE165303, GSE57345, and GSE145154) as training and validation sets for model development and evaluation. Log_2_-transformation was applied to the four datasets for data normalization prior to analysis. Last, for each model, the area under the ROC curve (AUC) value was calculated by using the pROC (version 1.18.5) package. The model with the highest average AUC in the training and testing datasets was considered optimal.

### Bulk transcriptome data analysis

We downloaded the bulk transcriptome datasets from Gene Expression Omnibus (GEO) containing patients with heart failure and healthy donors, accessed by GSE141090, GSE165303, GSE57345, and GSE145154 (S1 Table). Wilcoxon rank-sum test was used to compare the differential expression of genes of interest between heart failure and healthy groups. Pearson correlation analysis was employed to calculate the expression relationship between two genes. A *P*-value less than 0.05 was considered statistically significant. A correlation coefficient greater than 0 indicated a positive correlation between two genes, otherwise, they were negatively correlated.

### High-dimensional weighted gene co-expression network analysis (hdWGCNA)

We employed hdWGCNA package (version 0.2.24) to identify co-expression networks related to neuronal subsets. By selecting genes expressed in at least 10% of cells, we first used the K-nearest neighbor algorithm to calculate the similarity among cell populations. Next, according to the TestSoftPowers function, we set the soft threshold to nine and used it to construct the co-expression network. Lastly, genes with high connectivity in each module were displayed by using the GetModules function.

### Gene set enrichment analysis

The gene set enrichment score in each sample was evaluated by using ssGSEA method from the GSVA (version 1.48.3) package. Here, we calculated the enrichment score of 66 genes included in the predictive model on GSE165303, GSE57345, GSE141910 and GSE145154, respectively. Wilcoxon rank-sum test was employed to compare scores between heart failure and healthy groups.

## Results

### Landscape of neurons in heart failure

**Fig 1:**
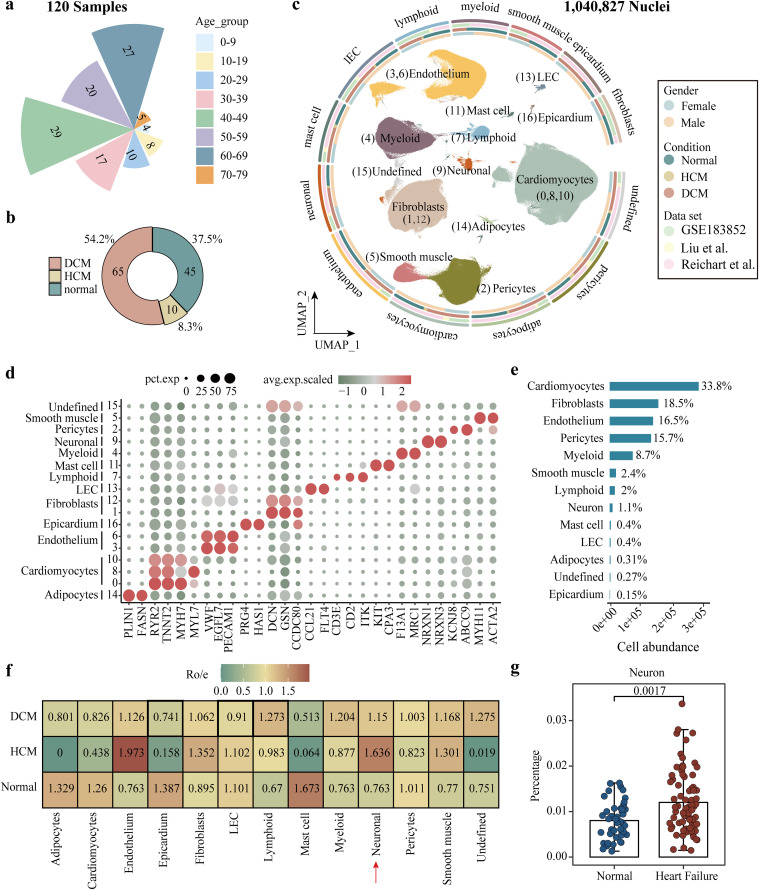
Single-nuclei profiling of human heart. **(a, b)** Distribution of age groups (a) and different conditions (b) among samples in the integrated data. **(c)** Annotation of cell types in the integrated data. **(d)** Expressions of marker genes derived from distinct cell types among cell clusters. **(e)** Statistics on the abundance of each cell type. **(f)** Condition preference of each cell type evaluated by the Ro/e index. **(g)** The difference in the proportion of neurons between the heart failure group and the normal control group. Wilcoxon rank-sum test was used. LEC, lymphatic endothelial cell.

We integrated large-scale snRNA-seq datasets from three human heart tissue cohorts, Reichart *et al.*, Liu *et al.*, and GSE183852 (S1 Table), and eliminated their batch effects by using Harmony algorithm (S1a and S1b Fig). A quality-controlled heart atlas was then constructed, containing 1,040,827 nuclei derived from 120 individuals throughout the whole lifespan (Fig 1a and 1b). These samples have a higher proportion of middle-aged and older people over 40 years old (Fig 1a). Unsupervised clustering identified 17 clusters within the map and defined 12 typical cell types based on marker genes obtained from the work of Koenig *et al.* [13] (Fig 1c and 1d)*.* Among them, neuronal cells exhibited high expression of canonical markers *NRXN1* and *NRXN3*. Meanwhile, we found the number of integrated neuronal cells reached 11,026, accounting for 1.1% of heart components (Fig 1e). Furthermore, based on calculating the Ro/e value, we discovered that the abundance of neuronal cells in DCM and HCM was both higher than that in healthy controls (Fig 1f) [25]. Notably, heart failure group showed a significantly higher proportion of neurons than that in healthy group (Fig 1g).

To explore the relationship between neuronal abundance and clinical performance, we collected several cardiac function indicators for analysis (S1c Fig). We found that the percentage of neuronal cells did not exhibit significant correlations with clinical features (S1d Fig). However, patients with preserved ejection fraction (left ventricular ejection fraction [LVEF] > 50%) showed a higher proportion of neuronal cells compared to those with reduced ejection fraction (LVEF < 50%) (S1e Fig). Additionally, we found strong heterogeneity within neurons by performing unsupervised clustering on 11,026 neuronal cells (S1f Fig). In this case, neuronal cells were clustered into ten clusters with distinct spatial boundaries (S1g Fig). In conclusion, the results demonstrate that specific characteristics among neuronal subsets are worthy of further exploration in heart failure.

### Depicting the properties of neuronal subsets in the pathogenesis of heart failure

**Fig 2:**
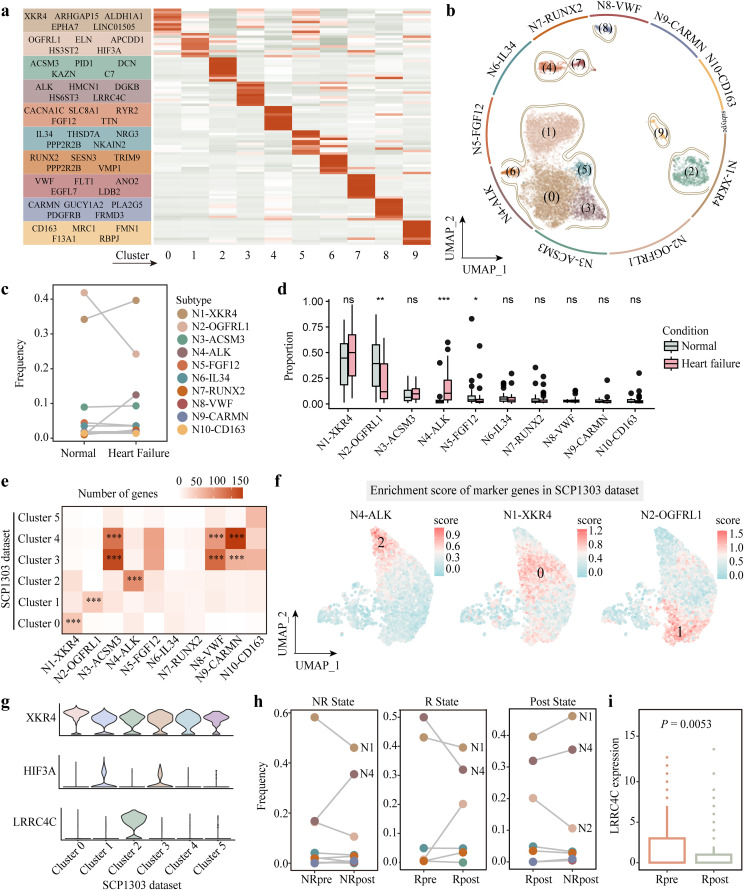
Identification of neuronal subsets associated with heart failure. **(a)** The heatmap displaying the top five differentially expressed genes in each neuronal cluster. **(b)** Definition of neuronal subsets. **(c)** Differences in the frequency of each neuronal subset between heart failure and healthy groups. **(d)** Comparisons upon the percentage of neuronal subsets between heart failure patients and healthy donors. **(e)** Overlapping of differential genes in neuronal subsets between our generated data and SCP1303 dataset. Hypergeometric test was used. **(f)** The enrichment scores of N1-XKR4, N2-OGFRL1, and N4-ALK marker genes in the SCP1303 dataset. **(g)** The expression of marker genes from N4-ALK, N1-XKR4, and N2-OGFRL1 over neuronal clusters in the dataset of SCP1303. **(h)** *Left* Changes in the frequency of neuronal subsets before and after receiving LVAD implantation in patients who did not respond to the treatment. *Center* Changes in the frequency of neuronal subsets in response to LVAD before and after receiving the treatment. *Right* Changes in the frequency of neuronal subsets at the statuses of responsive and unresponsive after receiving LVAD implantation. **(i)** The differential expression of *LRRC4C* in response to LVAD before and after receiving the implantation. *P < 0.05, **P < 0.01, ***P < 0.001, ns, no significance. In (d) and (i), *P* value was calculated by Wilcoxon rank-sum test. R or NR state, LVAD responsive or unresponsive status; Post State, after receiving LVAD implantation; R or NR pre, before LVAD implantation for treating individuals with or without response; R or NR post, after LVAD implantation for individuals with or without response.

Based on the neuronal subsets identified above, we further characterized their differences by comparing their compositions and molecular features in the heart. At first, we identified a set of cluster-specific marker genes by detecting significant differential expression within each cluster relative to all remaining clusters (logFC > 0.25, BH-adjusted *P*-value < 0.05). Subsequently, neuronal subsets were annotated by selecting a representative marker with high relative abundance from the top five differential expression genes (Fig 2a). This analysis determined ten distinct neuronal subsets, including N1-XKR4 (cluster 0), N2-OGFRL1 (cluster 1), N3-ACSM3 (cluster 2), N4-ALK (cluster 3), N5-FGF12 (cluster 4), N6-IL34 (cluster 5), N7-RUNX2 (cluster 6), N8-VWF (cluster 7), N9-CARMN (cluster 8), N10-CD163 (cluster 9) (Fig 2b). Compared with the normal heart, we observed that the failing heart exhibited a higher proportion of N1-XKR4 and N4-ALK subsets, and a lower proportion of N2-OGFRL1 subset (Fig 2c). We observed a significant increase in the proportion of N4-ALK subset, accompanied by a decrease in N2-OGFRL1 and N5-FGF12 subsets in heart failure patients compared to healthy controls (Fig 2d). Subsequently, we validated the identified neuronal subsets using an independent snRNA-seq dataset, SCP1303 [26], comprising samples from 11 DCM patients, 15 HCM patients, and 16 healthy donors (S1 Table and S2a-c Fig). After cell type annotation, extracting neuronal cells, and performing unsupervised clustering, we acquired six neuronal clusters (S2d Fig). We then identified differentially expressed genes for each cluster and calculated the similarity of paired clusters between the integrated map and SCP1303 cohort based on their overlapped marker genes (S2e Fig). The result displayed N1-XKR4 and Cluster 0, N2-OGFRL1 and Cluster 1, N4-ALK and Cluster 2 harbored the significantly largest number of overlapping marker genes, respectively (Fig 2e). Correspondingly, the enrichment scores of marker genes, derived from N1-XKR4, N2-OGFRL1, and N4-ALK, tended to be higher in their corresponding clusters in SCP1303 dataset (Fig 2f). Marker genes *XKR4* (N1-XKR4), *HIF3A* (N2-OGFRL1), and *LRRC4C* (N4-ALK) were highly expressed in Cluster 0, Cluster 1, and Cluster 2, respectively (Fig 2g). Collectively, these results validated the reproducibility of N1-XKR4, N2-OGFRL1, and N4-ALK, which were the focus of subsequent analysis.

Furthermore, we employed additional snRNA-seq data from heart failure patients before and after receiving LVAD implantation, GSE226314 [27], to correlate the characteristics of neuron subsets with clinical treatment. In GSE226314, the LVAD-responder group was defined as patients whose ejection fraction increased to ≥40% following LVAD implantation. Then, after data processing and neuronal clustering, we first mapped neuronal subsets markers identified previously to the processed neuronal profile (S3a Fig). The frequency of N4-ALK was increased in patients who did not benefit from LVAD implantation (Fig 2h, left panel). In contrast, the increase of N2-OGFRL1 and the decrease of N4-ALK were both related to the benefits of LVAD implantation therapy (Fig 2h, center and right panels). These results indicated that changes in the number of neurons were closely related to the outcome of LVAD implantation. And then, N4-ALK marker gene *LRRC4C* was found to be significantly downregulated in the LVAD responsive group and was upregulated in the group of patients who cannot benefit from LVAD implantation (Figs 2i and S3b). High *ALK* expression levels were associated with the LVAD unresponsive group (S3c Fig). In summary, our results reveal an elevated proportion of N4-ALK subset in heart failure and underscore the clinical significance of its reduction in relation to beneficial outcomes post-LVAD implantation.

### Transcriptional characteristics of failing heart-associated neuronal cells

**Fig 3:**
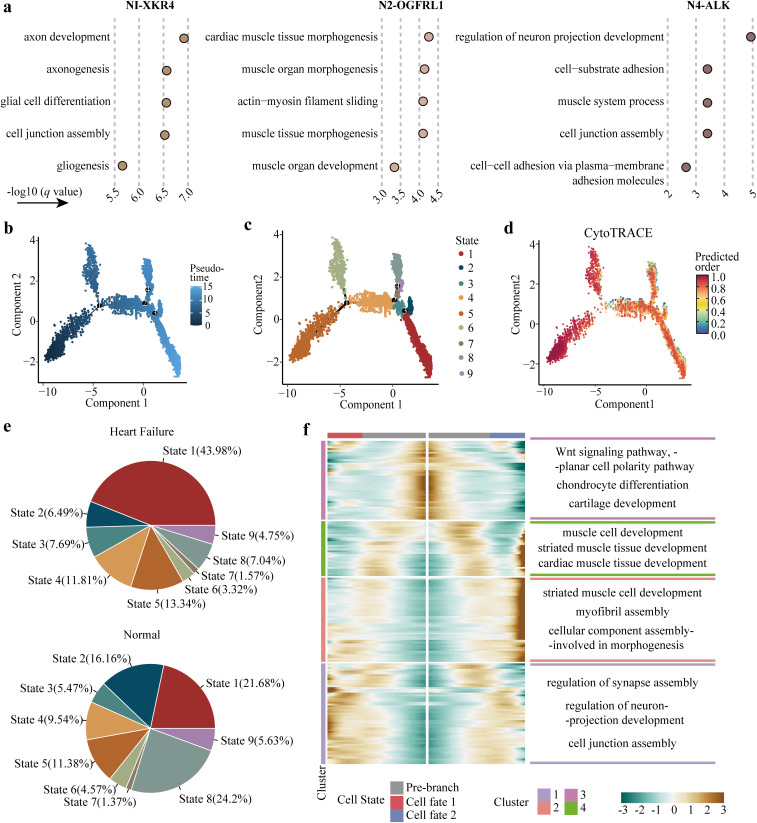
The transcriptional heterogeneity of neuronal subsets. **(a)** GO enrichment analysis of differentially expressed genes in N1-XKR4, N2-OGFRL1, and N4-ALK, respectively. The *q* value represented *P* value adjusted by Benjamini & Hochberg (BH). **(b)** Pseudo time analysis of reshaping neuronal cells over time. **(c)** Different cellular states divided by four branch nodes. **(d)** Evaluation of the differentiation ability on each neuronal cell. The color ranging from blue to red represented an increase in differentiation ability. **(e)** The composition of cellular states in heart failure and healthy conditions respectively. **(f)** *Left* Molecular changes along the trajectory conducted by branch three. *Right* GO enrichment analysis on each gene cluster with showing the top three pathways.

On the basis of the heterogeneous performance of neuronal subsets discovered above, we analyzed the different transcriptional features among these cells. Firstly, GO pathway enrichment analysis revealed that the failing heart-associated subsets (N1-XKR4 and N4-ALK) significantly participated in axonogenesis and neuron development process, while the normal heart-enriched N2-OGFRL1 subset likely regulated cardiac morphogenesis (Fig 3a). Then, pseudo time analysis conducted by Monocle2 [28] confirmed that neuronal cells were completely separated on four independent branches and divided into nine cellular states (Figs 3b, 3c, and S4a). To determine the initial point of the trajectory, we used CytoTRACE algorithm [29] to predict the relative differentiation status of cells (Fig 3d). The results showed that N3-ACSM3 had the highest score of differentiation ability, while N1-XKR4, N2-OGFRL1, and N4-ALK had lower scores (S4b Fig). After determining the initial state, we discovered that N1-XKR4, N2-OGFRL1, and N4-ALK subsets tended to appear at the end of the trajectory (S4c Fig). Meanwhile, another single-cell fate inference algorithm, SlingShot [30], also predicted N4-ALK subset as the terminal state on the third differentiation trajectory, while N3-ACSM3 subset remained in the initial stage of differentiation (S4d and S4e Fig).

Further, we found that the proportion of state one accounted for the majority cells in failing heart and was higher than that in normal heart (Fig 3e). Whereas, state two was more enriched in the normal heart (Fig 3e). It was interesting that state one and state two were distributed at the two ends of the third branch point of the trajectory. Then, we identified 174 genes with differential expression resulting from the third branch along the trajectory, which were ultimately clustered into four modules. Module one tended to be highly expressed at the end of the trajectory differentiating into state one, and modules two and four were both related to the differentiation into state two (Fig 3f). We discovered that genes contained in module one were significantly enriched in pathways of synapse assembly and neuron projection development regulation, and genes in module two and four were associated with cardiac muscle tissue development (Fig 3f). As expected, *ALK* and *LRRC4C* expression were elevated along with the trajectory of differentiation into state one (S4f Fig). Taken together, the above results characterize N2-OGFRL1 and N4-ALK subsets as two differentiation outcomes in the neuronal lineage.

### Cellular communication between N4-ALK cells and fibroblasts in heart failure

**Fig 4:**
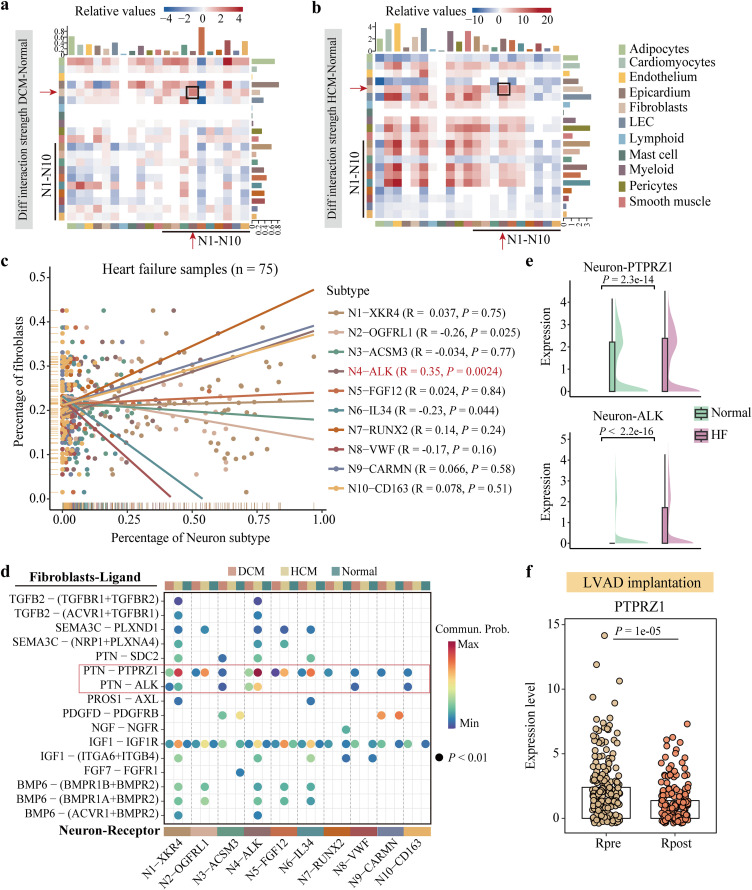
Cellular crosstalk between neurons and fibroblasts. **(a, b)** Differences in the strength of interactions between patients with DCM and healthy donors (a) and patients with HCM and healthy donors (b). The red arrow pointed out the cell types of N4-ALK on X-axis and fibroblasts in Y-axis. Black box marked the differential interaction between these two cell types. **(c)** Pearson correlation between the percentage of fibroblasts and the percentage of each neuronal subset in patients with heart failure. **(d)** Dot plot showing the communication between fibroblasts and neuronal subsets mediated by specific ligand-receptor pairs. **(e)** Differential expression of *PTPRZ1* and *ALK* in neurons between the heart failure group and the healthy group. **(f)** Differential expression of *PTPRZ1* before and after receiving implantation in the LVAD responsive group. In (e) and (f), Wilcoxon rank-sum test was used.

Considering the significant role of N4-ALK in heart failure, we hypothesized whether it promotes heart failure progression by interacting with neighboring cells. To examine this hypothesis, we performed cell-cell communication analysis between neurons and other cell types by using CellChat algorithm [31]. As a result, compared with the healthy group, we found that N4-ALK as “receiver” had more interactions with fibroblasts as “sender” both in DCM and HCM groups (Fig 4a and 4b). In addition, the frequency of fibroblasts was positively correlated with N4-ALK, but negatively correlated with N2-OGFRL1 (Fig 4c). It further supported the co-occurrence of fibroblasts and N4-ALK in the failing heart.

Furthermore, we found that the ligand PTN secreted by fibroblasts was most likely to bind to the N4-ALK receptor ALK or PTPRZ1 for communications (Fig 4d). The expression of *PTN* had great abundance in cardiac fibroblasts, especially in the heart failure group (S5a Fig). *ALK*, as a marker of N4-ALK, had a significant overexpression in the heart failure group compared to healthy group (Fig 4e). *PTPRZ1* was also highly expressed in N4-ALK subset and significantly up-regulated in heart failure group (Fig 4e). Then, spatial images of HCM patients showed that some spots co-expressed *PTN* and *PTPRZ1*, but almost none of them co-expressed *PTN* and *ALK* (S5b Fig). The PTN-PTPRZ1 axis was further validated using NicheNet [32]. This analysis confirmed that the PTN-PTPRZ1 pair represents a robust and relatively highly ranked interaction, exhibiting a high prior interaction weight (S5c Fig). Collectively, we proposed a close communication between fibroblasts and N4-ALK occurred through the PTN-PTPRZ1 axis. Regarding *PTPRZ1*, its expression was significantly downregulated in response to LVAD after receiving the implantation (Fig 4f). However, in patients not responding to LVAD implantation, *PTPRZ1* expression showed no significant change before and after treatment (S5d Fig).

### Identification of key upstream molecule regulating N4-ALK function

**Fig 5:**
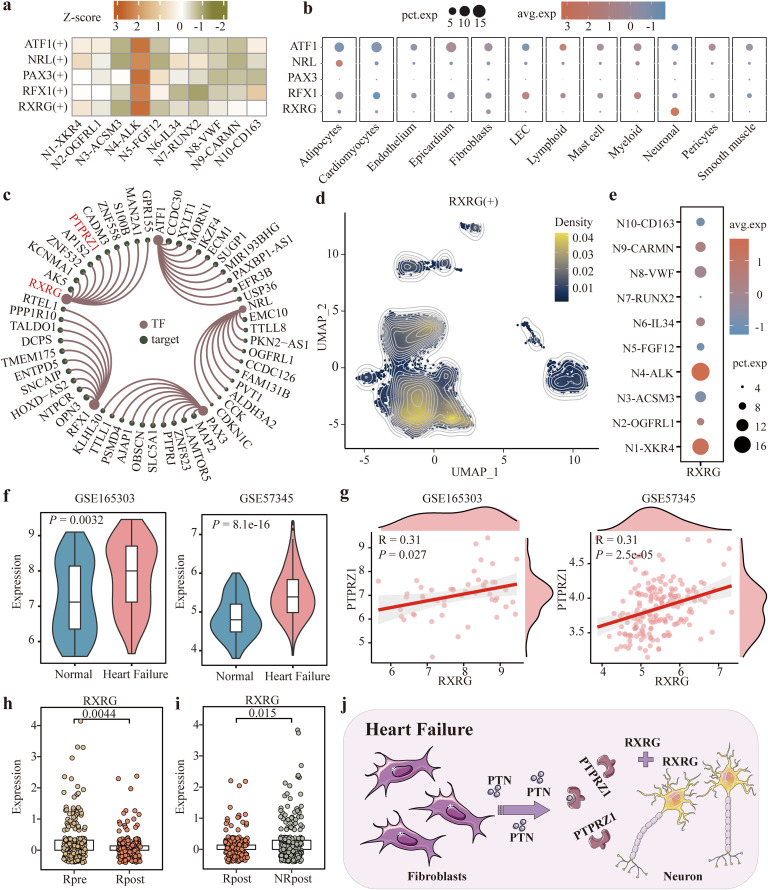
Identifying the transcriptional regulatory mechanism of neuronal cell in heart failure. **(a)** Five regulons with higher transcriptional activity in N4-ALK cells. **(b)** Differential expression of transcription factors *ATF1*, *NRL*, *PAX3*, *RFX1*, and *RXRG* in different cell types. **(c)** Transcriptional networks consisting of the five transcription factors and their top ten targets. **(d)** The density plot showing the enriched regions of RXRG regulon activity on the neuronal map. **(e)** The expression of *RXRG* across neuronal subsets. **(f)** Differential expression of *RXRG* in heart tissue between heart failure group and healthy group both in GSE165303 and GSE57345. **(g)** Pearson correlation between the expression of *RXRG* and *PTPRZ1* both in GSE165303 and GSE57345. **(h)** Differential expression of *RXRG* in response to LVAD before and after receiving LVAD implantation. **(i)** Differential expression of *RXRG* in cardiac neurons with or without response to LVAD after receiving implantation. **(j)** The crosstalk between cardiac neurons and fibroblasts mediated by overexpression of *RXRG* in heart failure. Images provided by Servier Medical Art (https://smart.servier.com), licensed under CC BY 4.0 (https://creativecommons.org/licenses/by/4.0/). In **(f)**, **(h)**, and **(i)**, Wilcoxon rank-sum test was used.

To gain certain regulatory relationship associated with N4-ALK, we performed single-cell regulatory network inference analysis using pySCENIC [33,34]. The heat map showed that regulons ATF1, NRL, PAX3, RFX1 and RXRG were distinctly expressed in N4-ALK (Figs 5a and S6a). Notably, except for *PAX3* with a low expression across cell types, only the transcription factor *RXRG* was highly expressed in neuronal cells. The other factors *ATF1*, *NRL*, *andRFX1* exhibited higher expression in lymphocytes, adipocytes, myeloid cells, respectively (Fig 5b). Through their regulatory networks, *PTPRZ1*, as a neuronal cell receptor of interest, was found to be involved in the RXRG regulatory network (Fig 5c). To explore RXRG’s regulatory role in N4-ALK, we extracted the top 50 genes targeted by *RXRG* and found 13 N4-ALK marker genes contained, such as *ALK* and *LRRC4C* (S6b Fig). The density plot also displayed that RXRG regulon exhibited higher density score at the N4-ALK and N1-XKR4 subsets (Fig 5d). Additionally, the expression of *RXRG* displayed strong enrichment in these two subsets (Fig 5e).

Furthermore, we found *RXRG* was significantly upregulated in the heart failure group compared to that in the healthy group in GSE165303 and GSE57345, and so was its target *PTPRZ1* (Figs 5f and S6c). Moreover, the levels of *RXRG* and *PTPRZ1* exhibited a significantly positive correlation (Fig 5g). Similar results were discovered in GSE141910 cohort (S6d Fig). But in GSE145154 dataset, *PTPRZ1* did not show differential expression between the two groups and had no significant correlation with *RXRG* (S6e Fig). Interestingly, *RXRG* expression was significantly reduced in LVAD-responsive group and upregulated in non-responsive patients (Fig 5h and 5i). There was relatively little association between *RXRG* expression and LVAD unresponsive status (S6f Fig). Additionally, *PTPRZ1* expression was significantly lower in the RXRG_low group than in the RXRG_high group (S6g Fig), and expression levels of *RXRG* and *PTPRZ1* exhibited a significant positive correlation (S6h Fig). Two online tools, FIMO (https://meme-suite.org/meme/tools/fimo) and JASPAR (https://jaspar.elixir.no/), consistently identified putative RXRG motif-binding sites in the 2,000-bp region upstream of the PTPRZ1 transcription start site (S2 Table), further confirming a potential regulatory relationship between *RXRG* and *PTPRZ1*. In summary, heart failure was associated with increased *RXRG* expression in cardiac neurons, which upregulates its target *PTPRZ1*, potentially facilitating crosstalk with fibroblasts via the ligand PTN (Fig 5j).

### Validation of the mechanism mediated by N4-ALK cells in heart failure

**Fig 6:**
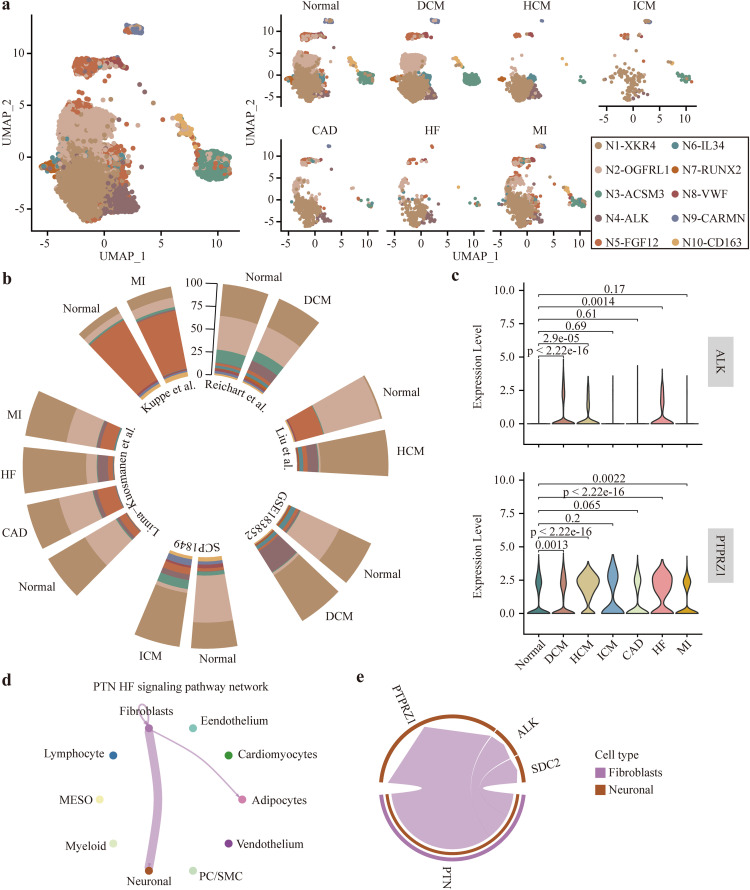
Analysis of the molecular mechanism mediated by the newly generated neuronal subsets. **(a)** Label transfer learning analysis on the newly generated neuronal map included different etiologies. **(b)** Differences of the proportion in neuronal subsets across different datasets. **(c)** The differential expression of *PTPRZ1* and *ALK* between normal heart and diseased heart induced by various etiologies. Wilcoxon rank-sum test was used. **(d)** Cell-cell communications of PTN signaling pathway. **(e)** Pairs of ligand and receptor in the interactions between fibroblasts and neurons in the failing heart.

Although the working mechanism of N4-ALK has been elucidated previously, it still needs further validation and applicability assessment. For this purpose, we additionally integrated data from three research works, SCP1849, Linna-Kuosmanen *et al.*, and Kuppe *et al.* (S1 Table) [35–37], and included patients with various cardiac conditions, namely ischemic cardiomyopathy (ICM), ischemic heart failure (IHF), non-ischemic heart failure (NIHF), coronary atherosclerosis (CAD), myocardial infarct (MI). By extracting neuronal cells from these data sets, we eventually obtained 213, 439, 32, 1,001, 2,997 neuronal cells derived from ICM, IHF, NIHF, CAD, and MI, respectively. In view of the lower abundance of neurons in NIHF, we combined IHF and NIHF into the heart failure group, named newly added heart failure group. Subsequently, we transferred the integrated cardiac neuronal subsets labels into these newly added cells (Fig 6a). By this way, among the newly integrated map, neuronal subsets derived from same data set had higher co-expression intensity (S7a Fig). Consistent with above findings, we found that the percentage of N4-ALK was also higher in the newly added heart failure group compared to that in control group (Fig 6b). The abundance of N5-FGF12 was higher in MI and CAD groups in the work of Kuosmanen *et al.* Whereas, in the work of Kuppe *et al.*, N5-FGF12 did not show a significant difference in proportion between MI and normal control groups (Fig 6b).

Consistently, we found that N4-ALK marker genes *PTPRZ1*, *ALK*, and *LRRC4C* were significantly upregulated in DCM, HCM, ICM, and heart failure groups (Figs 6c and S7b). Compared with the control group, *RXRG* had a significant over-expression in HCM and ICM, but appeared to be down-regulated in DCM (S7c Fig). We reasonably suspected that the deviation might be due to an excessive number of neurons in DCM. To verify this, we examined the distribution of neuronal cells in SCP1303, where the number of neurons was balanced among HCM (n = 1,528), DCM (n = 1,337), and normal control (n = 1,409). Actually, the dot plot showed that *RXRG* did have a higher expression in DCM and HCM rather than in controls (S7d Fig).

Lastly, we discovered that in this heart failure group, neuronal cells engaged in more communications to activate PTN signaling pathway, which was scarcely present in the healthy state (S8a Fig). In PTN-mediated cellular communications, neuronal cells predominantly functioned as “receiver”, while fibroblasts mainly served as “senders”, thereby contributing most to the crosstalk (Figs 6d and S8b). It was not surprising to observe that the PTN-PTPRZ1 axis showed the highest interaction strength in the heart failure group (Fig 6e). Moreover, the activation of PTN-PTPRZ1 axis only existed in the communication between fibroblasts (rather than other cell types) and neuronal cells in heart failure (S8c Fig). In summary, results derived from validation sets suggested stable interaction between fibroblasts and neurons via the PTN-PTPRZ1 axis in heart failure patients.

### Developing a molecular model to predict heart failure patients by machine learning algorithms

**Fig 7:**
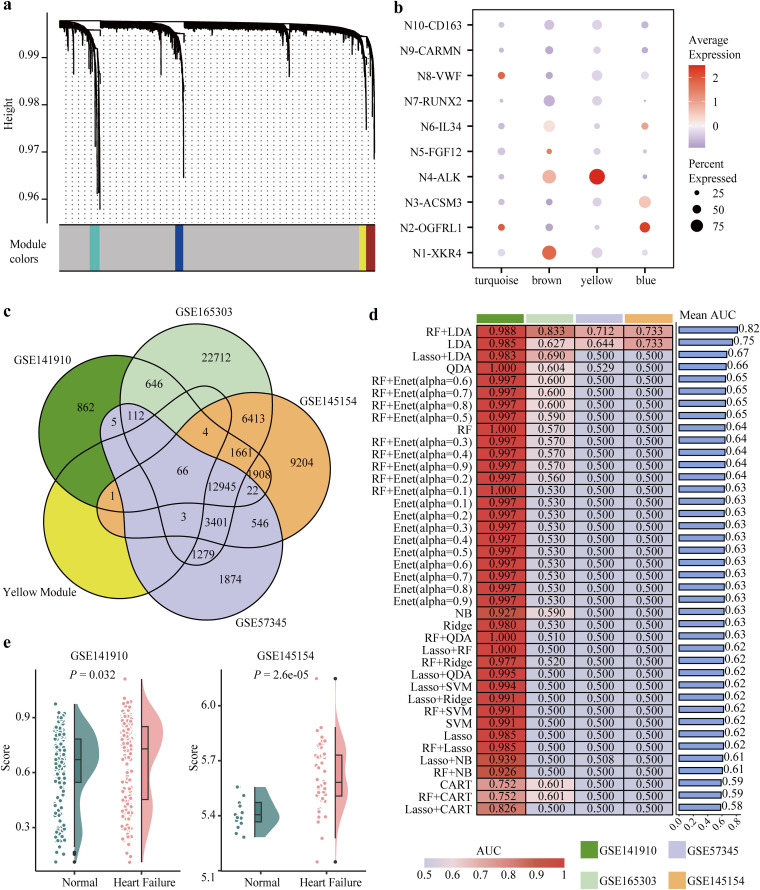
Construction of a predictive model for heart failure using N4-ALK associated features. **(a)** Identification of co-expressed gene modules within neurons. **(b)** The scores of four gene modules in ten subsets of neurons. **(c)** Overlapping genes of “yellow” module and genes detected in data sets GSE141910, GSE165303, GSE145154, and GSE57345. **(d)** Heat map displaying the AUC score of each heart failure prediction model, and bar plot illustrating its average AUC score across four datasets. **(e)** The differential enrichment scores of 66 genes between the heart failure group and the healthy group in GSE141910 and GSE145154, respectively. Wilcoxon rank-sum test was used. AUC, area under the ROC curve.

To better link neuronal characteristics to clinical applications, a molecular model was both essential and feasible. A neuron weighted co-expression network was constructed using the hdWGCNA algorithm [38], and four functional co-expression modules were identified (Figs 7a and S9a). Among them, the “yellow” module contains 74 genes, including N4-ALK marker genes *HCMCN1* and *DGKB* (S9b Fig). Moreover, genes within the “yellow” module were highly expressed in N4-ALK but not in other neuronal subsets, suggesting a close association with N4-ALK (Figs 7b and S9c). Thus, we overlapped these 74 genes with the genes detected in the profiles of four data cohorts and eventually acquired 66 genes for subsequent analysis (Fig 7c). By using nine machine learning algorithms, 40 combined prediction methods were constructed according to the LOOCV framework [24]. Finally, as shown in Fig 7d, the combined model of RF and LDA exhibited the optimal performance (average AUC = 0.82), followed by using LDA alone (average AUC = 0.75). The optimal model exhibited consistently high sensitivity across the three datasets, while its specificity was higher in the GSE57345 dataset (S10a Fig). The optimal model consisted of 56 genes with importance scores greater than one, including *PTPRZ1* and other candidate genes (S10b Fig). To note, the classification performance of *NPPB* expression, a gene closely linked to heart failure, was consistently inferior to our established RF plus LDA model across all four datasets (S10c Fig). In summary, based on N4-ALK subset-associated features, we developed a heart failure prediction model by combining RF-based feature selection and LDA-driven classification and prediction.

Furthermore, when applying the ssGSEA algorithm to quantify N4-ALK subset-related enrichment scores, we consistently observed significantly higher scores in the heart failure group than in the healthy donors across four data cohorts, confirming the strong correlation between N4-ALK subset features and heart failure (Figs 7e and S9d).

## Discussion

In this study, we constructed a map of neuronal cells in the heart, which consisted of 11,026 cells. The landscape of cardiac neurons enabled a systematic depiction of the specific compositions, transcriptional heterogeneity, and developmental dynamics among neuronal subsets. The strong heterogeneity of neuronal cells underscores the importance of further characterizing their specific features.

In this work, we integrated snRNA-seq datasets to enhance neuronal cell abundance, thereby identifying ten distinct subsets, and validation using an independent dataset further confirmed their stable and reproducible classification. Compared with neuronal subsets identified in healthy heart conducted by Litviňuková *et al.* [12], we identified notable similarities between our defined subsets and the neuronal clusters reported in their study, such as N8-VWF and NC3 (co-express typical endothelial cells genes), N3-ACSM3 and NC2 (co-express typical fibroblast genes), N5-FGF12 and NC4 (co-express typical cardiomyocyte genes) (Fig 2a). On the other hand, Cui *et al.* studied the neuron heterogeneity in DCM patients and identified six neuronal subsets, which were named “Neurons 1” to “Neurons 6”, respectively [19]. When compared with their identified neuronal clusters, we found that our work covered nearly 30-fold more neurons than their study. Certainly, we also found some similarities between our determined neuronal types and those six subsets identified by Cui *et al.* For example, N4-ALK and “Neurons 1” both participate in some neuron-related pathways, such as axon guidance and neuron project development. Along the pseudo time, both types appear at the end of the neuronal differentiation trajectory.

In our work, we discovered that RXRG regulon has a higher activity in N4-ALK and it regulates multiple N4-ALK marker genes, especially *PTPRZ1* and *ALK*. With regard to *PTPRZ1*, it also acted as a receptor for N4-ALK and participated in crosstalk with fibroblasts in heart failure. Interestingly, compared to LVAD non-responders, the abundance of heart failure-related neuron subset (N4-ALK) and expression levels of its marker genes (*PTPRZ1* and *LRRC4C*) were reduced in LVAD responders. These observations suggest a potential functional link between the N4-ALK neuronal subset and the therapeutic response to LVAD. Of note, based on N4-ALK features, our machine learning model exhibited robust performance in classifying heart failure patients. For this model, the default classification threshold in LDA is governed by the maximum a posteriori probability criterion, potentially providing a simple, objective, and reproducible decision rule for clinical applications.

As highlighted in the review by Pahuja *et al.*, sympathetic nervous system upregulation links to heart failure outcomes, and neurohormonal activation correlates with symptom severity, functional decline, and mortality in heart failure [39]. This underscores the need to investigate neural regulatory mechanisms in heart failure, however, the precise molecular mechanisms underlying neuronal modulation remain undefined. In heart tissue, cardiac fibroblasts play a key role in regulating the development of the myocardial and vascular systems [40]. Plenty of investigations have reported functional crosstalk between fibroblasts and other cell types, such as cardiomyocytes and endothelial cells, contributes to diseased heart [41–44]. Conversely, few studies have focused the crosstalk signals between fibroblasts and neurons. Here, we identified a robust crosstalk between fibroblasts and N4-ALK cells. The consistent finding of the PTN-PTPRZ1 axis, confirmed by both the independent NicheNet prediction model and its presence in the newly analyzed heart failure cohort, strongly underscore its robustness and critical functional role in heart failure pathogenesis. Taken together, we herein proposed for the first time that fibroblasts may interact with N4-ALK cells to accelerate heart injury via the PTN-PTPRZ1 axis.

It has been widely reported that PTPRZ1-mediated signaling axes, such as PTN-PTPRZ1 and PTPRZ1-MET, have an important role in modulating tumor-associated macrophage polarization and regulating glioblastoma progression [45,46]. By blocking the PTN-PTPRZ1 signaling, it seems to reshape the immune microenvironment in glioblastoma and enhance its therapeutic efficacy [47]. In addition, Ma *et al.* demonstrated the overexpression of *PTPRZ1* is related to promoting malignancy of oral submucous fibrosis [48]. Whereas, limited research has been performed to correlate *PTPRZ1* with cardiac fibrosis. In our work, we first characterized *PTPRZ1* as a marker of N4-ALK cells and elucidated its interaction with PTN secreted by cardiac fibroblasts in heart failure. In contrast to our finding, Katraki-Pavlou *et al.* proposed that *PTPRZ1* is expressed in fetal heart rather than adults and loss of *PTPRZ1* leads to heart dysfunction using mouse and zebrafish models [49]. But we did confirm the high expression of *PTPRZ1* in the human heart failure group both at the cellular and population level across various cohorts.

There were some limitations in our current research. The low abundance of neuronal cells made it difficult to verify the existence of N4-ALK cells and their crosstalk with fibroblasts using high-throughput data. To this end, we figured that when the abundance of two cell types shows a significant positive correlation, it can also reflect their co-occurrence to a certain extent. In addition, spatial transcriptome imaging validated the colocalization of *PTN* and *PTPRZ1* in human heart tissues. While spots with their simultaneous high expression were relatively limited, this is likely due to the scarcity of PTPRZ1-expressing spots. Nevertheless, the robustness of the PTN-PTPRZ1 axis was independently corroborated by NicheNet analysis and cross-dataset validation. Lastly, although we have tried our best to verify our findings utilizing bioinformatics methods, it still needs to validate key neuronal regulatory factors and mechanisms in vitro and in vivo experiments in the future.

In conclusion, our study systematically integrates current resources of neurons across heart failure studies, providing a perspective on the heterogeneity of cardiac neurons. Our cardiac neurons blueprint can further enable the development of neuronal cell-associated treatment for heart failure patients.

## Supporting information

S1 Table Data resources and detailed information. (XLSX)

S2 Table Transcription factor binding sites were predicted using online tools. (XLSX)

S1 Fig Correlating the abundance of cell types with cardiac function. (a) UMAP plots illustrate the batch effect correction across different datasets before and after applying Harmony algorithm. (b) Unsupervised clustering analysis on the integrated data. (c) The distribution of cardiac indicators in patients with heart failure. LVEF, left ventricular ejection fraction; LVIDd, left ventricular internal diastolic dimensions; LVIDs, left ventricular internal systolic dimensions; BNP, brain natriuretic peptide; BMI, body mass index. (d) Calculation of the correlation between cell type abundance and cardiac indicators. *P < 0.05, **P < 0.01, ***P < 0.001, Pearson correlation analysis. (e) The difference in the percentage of neurons between patients with higher and lower ejection fractions. Wilcoxon rank-sum test was used. (f) Neuron integration across the three data sets derived from DCM, HCM, and healthy donors. (g) Unsupervised clustering analysis on the integrated neuronal map.
(TIF)

S2 Fig Verification of neuronal subsets in the SCP1303 dataset. (a) Unsupervised clustering analysis on all cells from SCP1303. (b) The expression of cell type markers among clusters. (c) Cell type annotation. (d) Unsupervised clustering analysis on neuronal cells based on UMAP algorithm. (e) The volcano plot displaying significantly overexpressed genes on each neuronal cluster. Text annotations showing the top five genes with the greatest differences in each cluster.
(TIF)

S3 Fig Label transfer of integrated neuronal subsets to GSE226314 dataset. (a) Neuronal label transferring in GSE226314. (b) *Left* Differential *LRRC4C* expression before and after receiving LVAD implantation in non-responders. *Right* Differential expression of *LRRC4C* between LVAD responders and LVAD unresponders. (c) *Left* Differential *ALK* expression in response to LVAD before and after receiving implantation. *Center* Differential *ALK* expression before and after receiving LVAD implantation in non-responders. *Right* The differential expression of *ALK* between LVAD responsive group and LVAD unresponsive group. In (b) and (c), Wilcoxon rank-sum test was used.
(TIF)

S4 Fig Analysis of differentiation trajectories conducted by neuronal subsets. (a) Distributions of different neuronal subsets along the trajectory. (b) The box plot exhibiting the differentiation ability of different neuronal subsets. (c) Differences in differentiation time of neuronal subsets. (d) Neuronal lineage differentiation inferred by Slingshot algorithm. (e) The neuronal differentiation result inferred by the third trajectory. (f) Changes of the expression of *ALK* and *LRRC4C* along the trajectory conducted by the third branch.
(TIF)

S5 Fig Validation of PTN-PTPRZ1 axis using spatial transcriptome data and NicheNet prediction. (a) Differential expression of *PTN*, *PTPRZ1*, and *ALK* in different cell types and conditions. (b) Co-expression of *PTN* and *PTPRZ1*, as well as *PTN* and *ALK*, in the spatial images of seven HCM patients. Red arrows point out spots of co-expression. (c) The heatmap visualizes the prior interaction weights of the top-ranked ligand-receptor pairs inferred by the NicheNet tool, filtered for pairs with a prior score > 0.5. Ligands (rows) are expressed in fibroblasts and receptors (columns) are expressed in N4-ALK subset cells. (d) *Left* The differential expression of *PTPRZ1* before and after receiving implantation in patients who did not respond to LVAD. *Right* The differential expression of *PTPRZ1* between LVAD responsive and unresponsive groups. Wilcoxon rank-sum test was used.
(TIF)

S6 Fig The regulatory role of RXRG in cardiac dysfunction. (a) The top five regulons with higher transcriptional activity in each neuronal subset. (b) The transcriptional network of *RXRG* with its top 50 targets. (c) Differential expression of *PTPRZ1* in heart tissue between heart failure group and healthy group using GSE165303 and GSE57345, respectively. (d, e) Differential expression of *RXRG* (*left*) and *PTPRZ1* (*median*) in heart tissue between heart failure group and healthy group in GSE141910 (d) and GSE145154 (e), respectively. *Right* Pearson correlation between the expression of *RXRG* and *PTPRZ1* in heart failure samples based on GSE141910 (d) and GSE145154 (e), respectively. (f) Differential expression of *RXRG* before and after receiving LVAD implantation in patients who did not benefit from implantation. (g) Differential expression counts of *PTPRZ1* between the RXRG_low (n = 6,538, cells with a zero count of RXRG) and RXRG_high (n = 831, cells with non-zero count of RXRG) neuronal cells from heart failure patients. (h) Pearson correlation analysis between *RXRG* and *PTPRZ1* expressions in heart failure-neuronal cells with non-zero counts values (n = 565). In (c), (d), (e), (f), and (g), except for right plots of (d) and (e), Wilcoxon rank-sum test was used.
(TIF)

S7 Fig Analysis of neuronal map. (a) Evaluation of similarity among neuronal subsets from different studies. (b, c) The differential expression of *LRRC4C* (b) and *RXRG* (c) between normal heart and diseased heart. Wilcoxon rank-sum test was used. (d) Distribution of the expression of *RXRG* in neuronal clusters from SCP1303.
(TIF)

S8 Fig Cellular communication of newly generated neuronal map. (a) The overall signaling of cell-cell communications in heart failure and healthy samples, respectively. (b) The communication strength involved in PTN signaling among cell types. (c) The communications between fibroblasts and other cell types in failing and healthy hearts.
(TIF)

S9 Fig High-dimensional weighted gene co-expression network analysis. (a) Determination of the soft power threshold of the hdWGCNA algorithm. (b) Bar charts display the weights of hub genes in each module and listed the top five genes. (c) The average score of “yellow” module genes in the neuronal subsets.
(TIF)

S10 Fig Analysis of heart failure prediction models. (a) Heat maps display the specificity and sensitivity scores of 40 heart failure predictive models across the four datasets. (b) Ranking of 66 identified genes based on importance scores from random forest feature selection, measured by the Mean Decrease Gini. (c) Evaluation of the predictive efficacy of *NPPB* expression for classifying heart failure patients. (d) The differential enrichment scores of 66 genes between the heart failure group and the healthy group in GSE165303 and GSE57345, respectively. Wilcoxon rank-sum test was used.
(TIF)
