# Supplementary figures and images for: Deciphering the cardiac neuron landscape in heart failure patients
Source: PLoS Comput Biol. 2026 Mar 20;22(3):e1014082. doi: 10.1371/journal.pcbi.1014082 (PMC13004344; doi:10.1371/journal.pcbi.1014082)

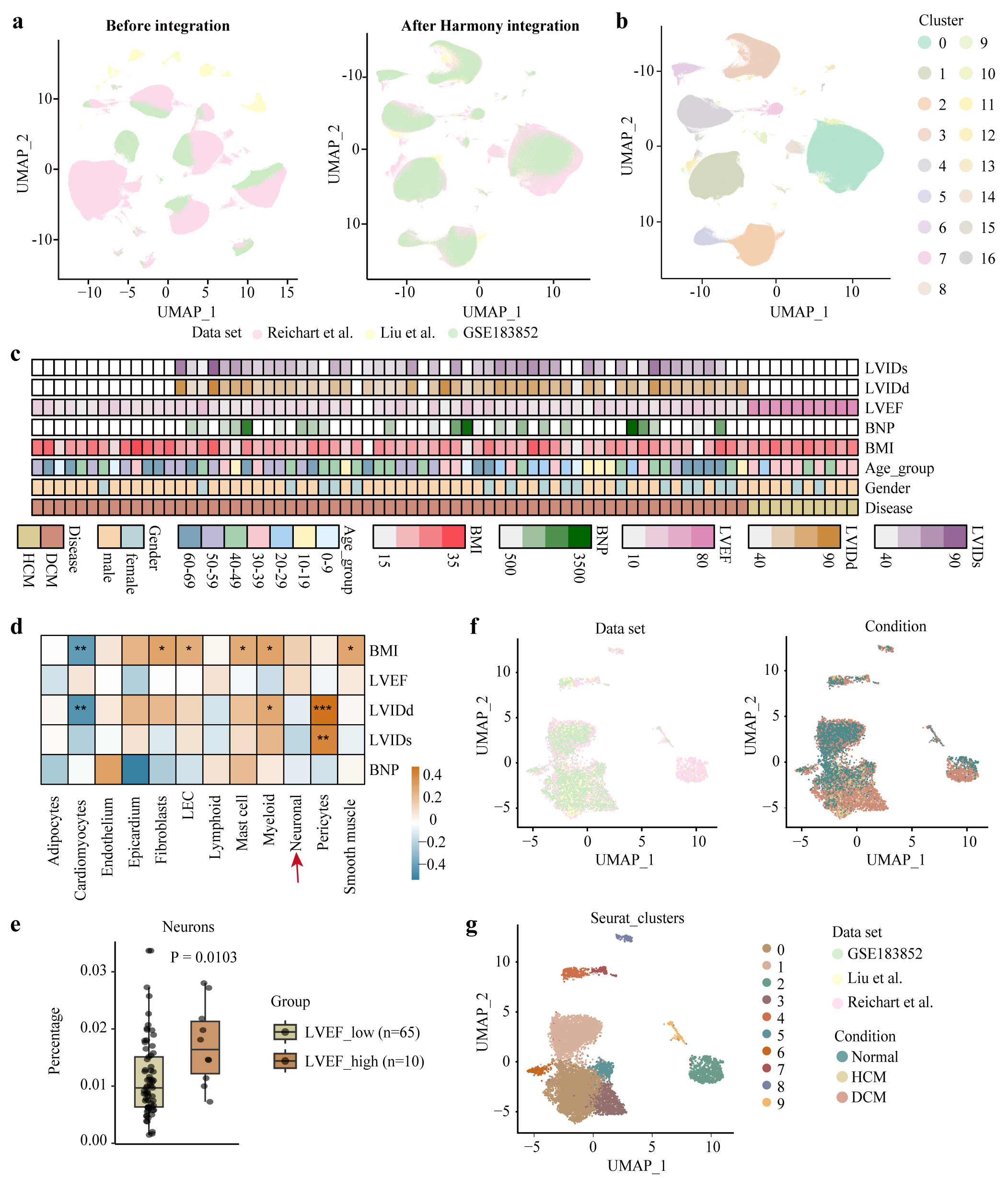

Supplement: S1 Fig — (a) UMAP plots illustrate the batch effect correction across different datasets before and after applying Harmony algorithm. (b) Unsupervised clustering analysis on the integrated data. (c) The distribution of cardiac indicators in patients with heart failure. LVEF, left ventricular ejection fraction; LVIDd, left ventricular internal diastolic dimensions; LVIDs, left ventricular internal systolic dimensions; BNP, brain natriuretic peptide; BMI, body mass index. (d) Calculation of the correlation between cell type abundance and cardiac indicators. *P < 0.05, **P < 0.01, ***P < 0.001, Pearson correlation analysis. (e) The difference in the percentage of neurons between patients with higher and lower ejection fractions. Wilcoxon rank-sum test was used. (f) Neuron integration across the three data sets derived from DCM, HCM, and healthy donors. (g) Unsupervised clustering analysis on the integrated neuronal map. (TIF) [file pcbi.1014082.s003.tif]

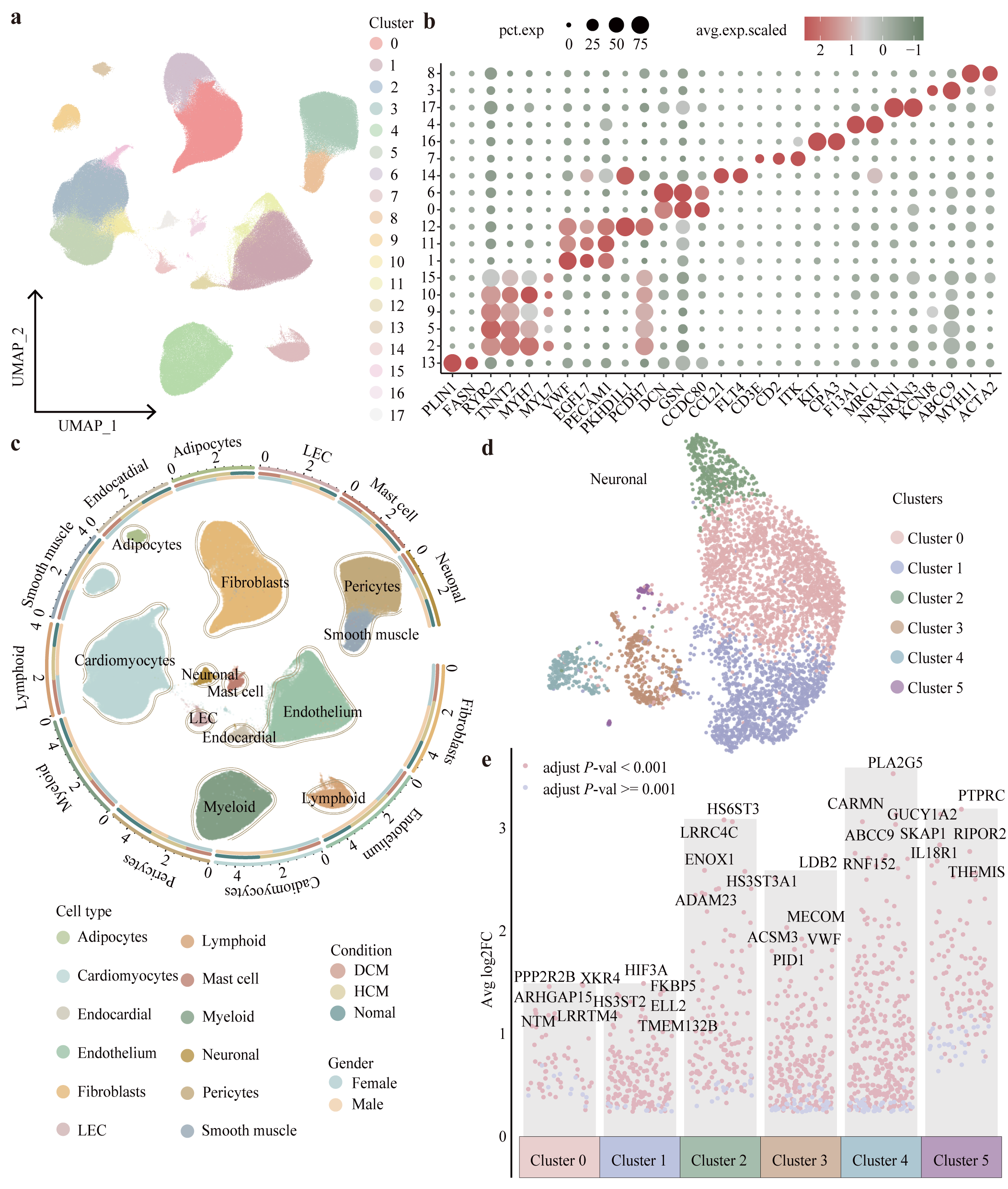

Supplement: S2 Fig — (a) Unsupervised clustering analysis on all cells from SCP1303. (b) The expression of cell type markers among clusters. (c) Cell type annotation. (d) Unsupervised clustering analysis on neuronal cells based on UMAP algorithm. (e) The volcano plot displaying significantly overexpressed genes on each neuronal cluster. Text annotations showing the top five genes with the greatest differences in each cluster. (TIF) [file pcbi.1014082.s004.tif]

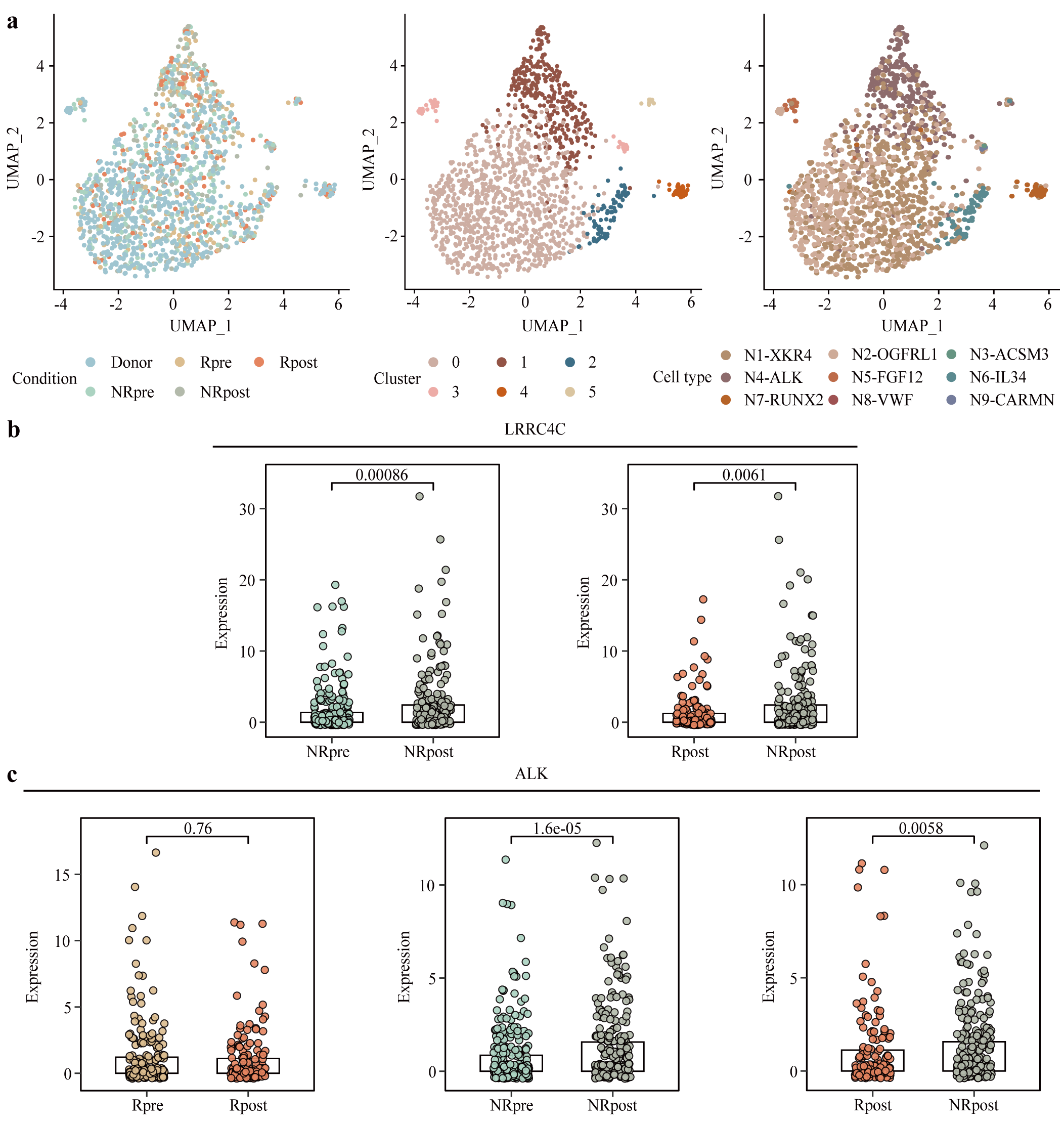

Supplement: S3 Fig — (a) Neuronal label transferring in GSE226314. (b) Left Differential LRRC4C expression before and after receiving LVAD implantation in non-responders. Right Differential expression of LRRC4C between LVAD responders and LVAD unresponders. (c) Left Differential ALK expression in response to LVAD before and after receiving implantation. Center Differential ALK expression before and after receiving LVAD implantation in non-responders. Right The differential expression of ALK between LVAD responsive group and LVAD unresponsive group. In (b) and (c), Wilcoxon rank-sum test was used. (TIF) [file pcbi.1014082.s005.tif]

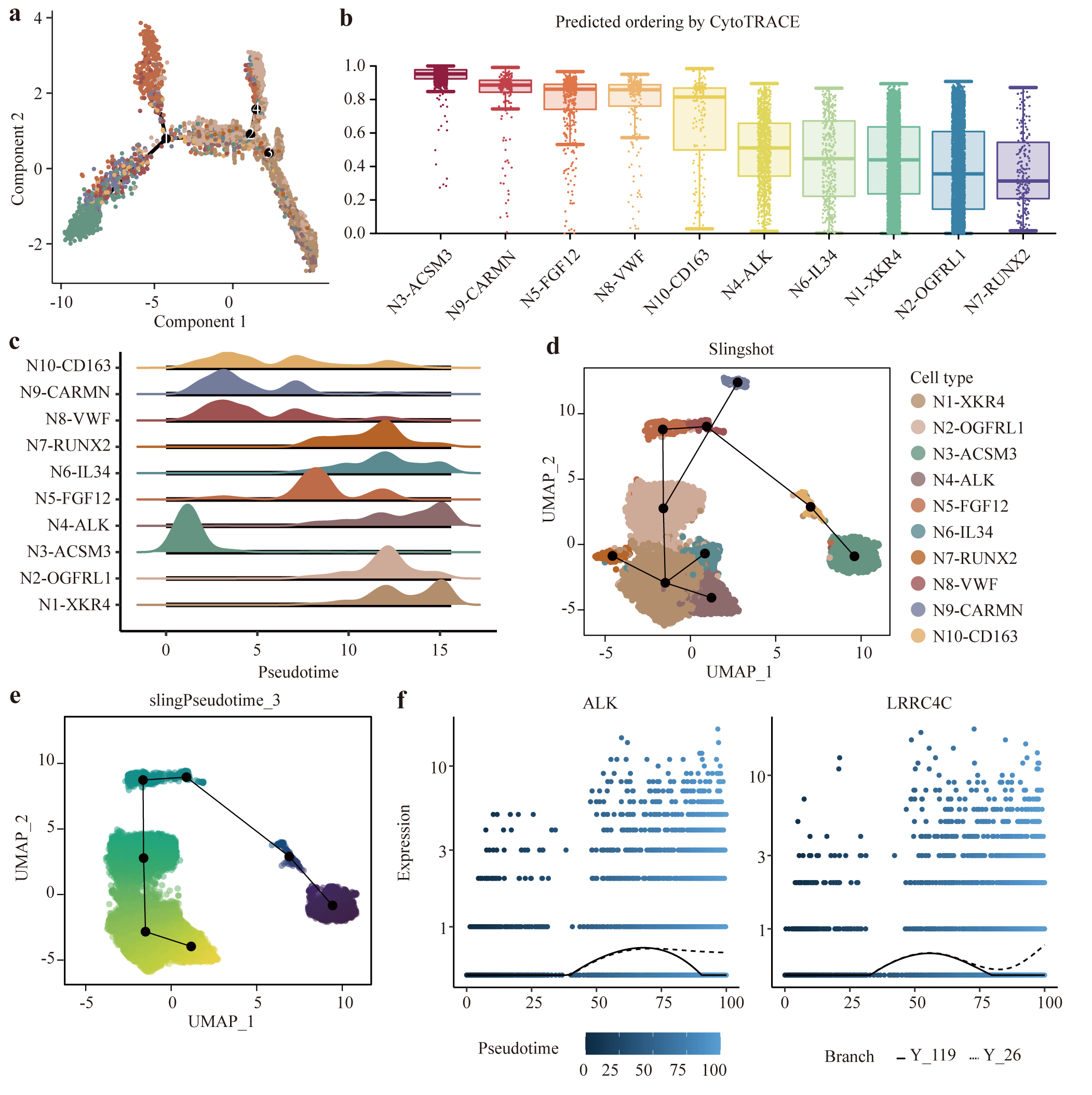

Supplement: S4 Fig — (a) Distributions of different neuronal subsets along the trajectory. (b) The box plot exhibiting the differentiation ability of different neuronal subsets. (c) Differences in differentiation time of neuronal subsets. (d) Neuronal lineage differentiation inferred by Slingshot algorithm. (e) The neuronal differentiation result inferred by the third trajectory. (f) Changes of the expression of ALK and LRRC4C along the trajectory conducted by the third branch. (TIF) [file pcbi.1014082.s006.tif]

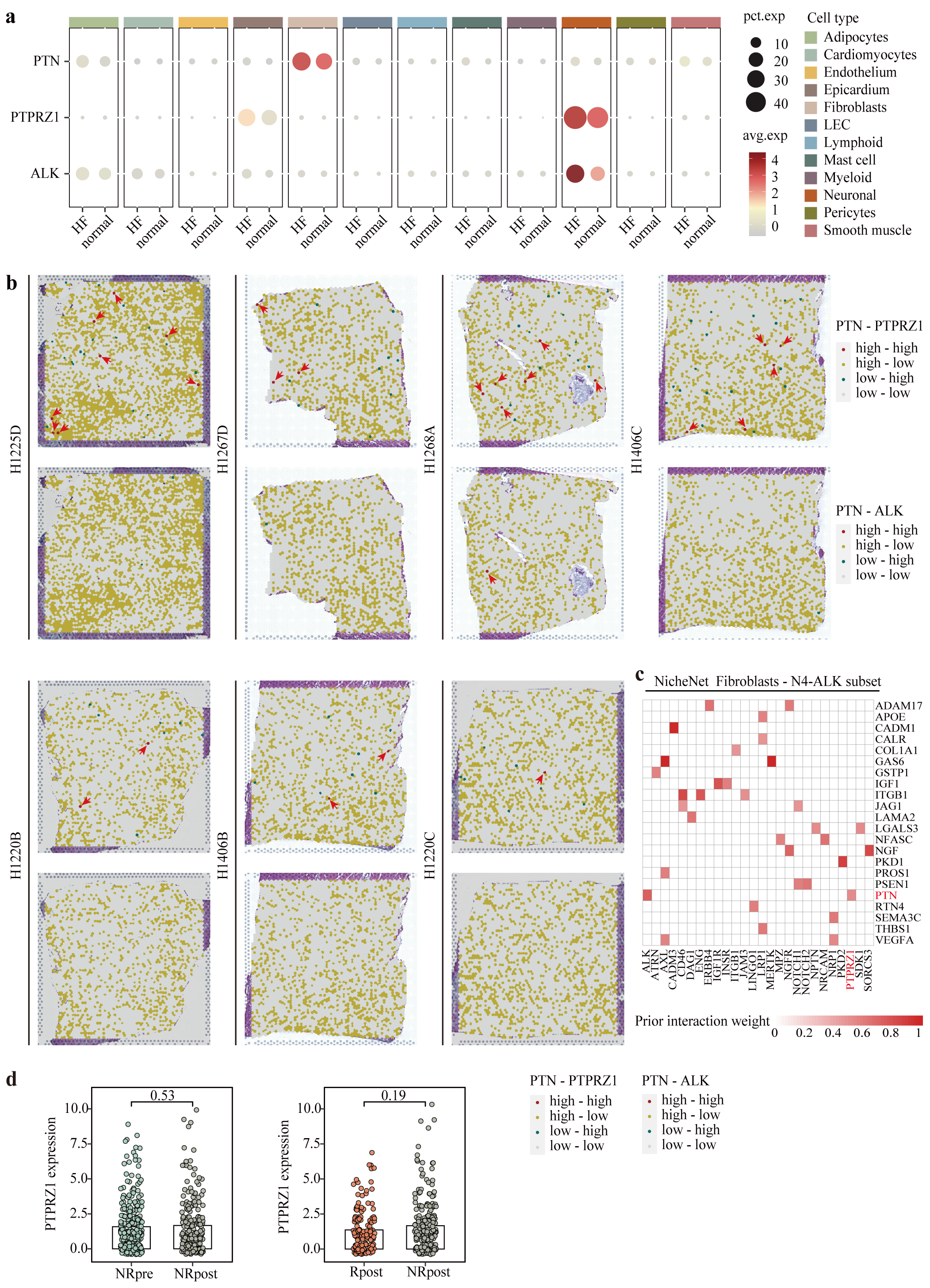

Supplement: S5 Fig — (a) Differential expression of PTN, PTPRZ1, and ALK in different cell types and conditions. (b) Co-expression of PTN and PTPRZ1, as well as PTN and ALK, in the spatial images of seven HCM patients. Red arrows point out spots of co-expression. (c) The heatmap visualizes the prior interaction weights of the top-ranked ligand-receptor pairs inferred by the NicheNet tool, filtered for pairs with a prior score > 0.5. Ligands (rows) are expressed in fibroblasts and receptors (columns) are expressed in N4-ALK subset cells. (d) Left The differential expression of PTPRZ1 before and after receiving implantation in patients who did not respond to LVAD. Right The differential expression of PTPRZ1 between LVAD responsive and unresponsive groups. Wilcoxon rank-sum test was used. (TIF) [file pcbi.1014082.s007.tif]

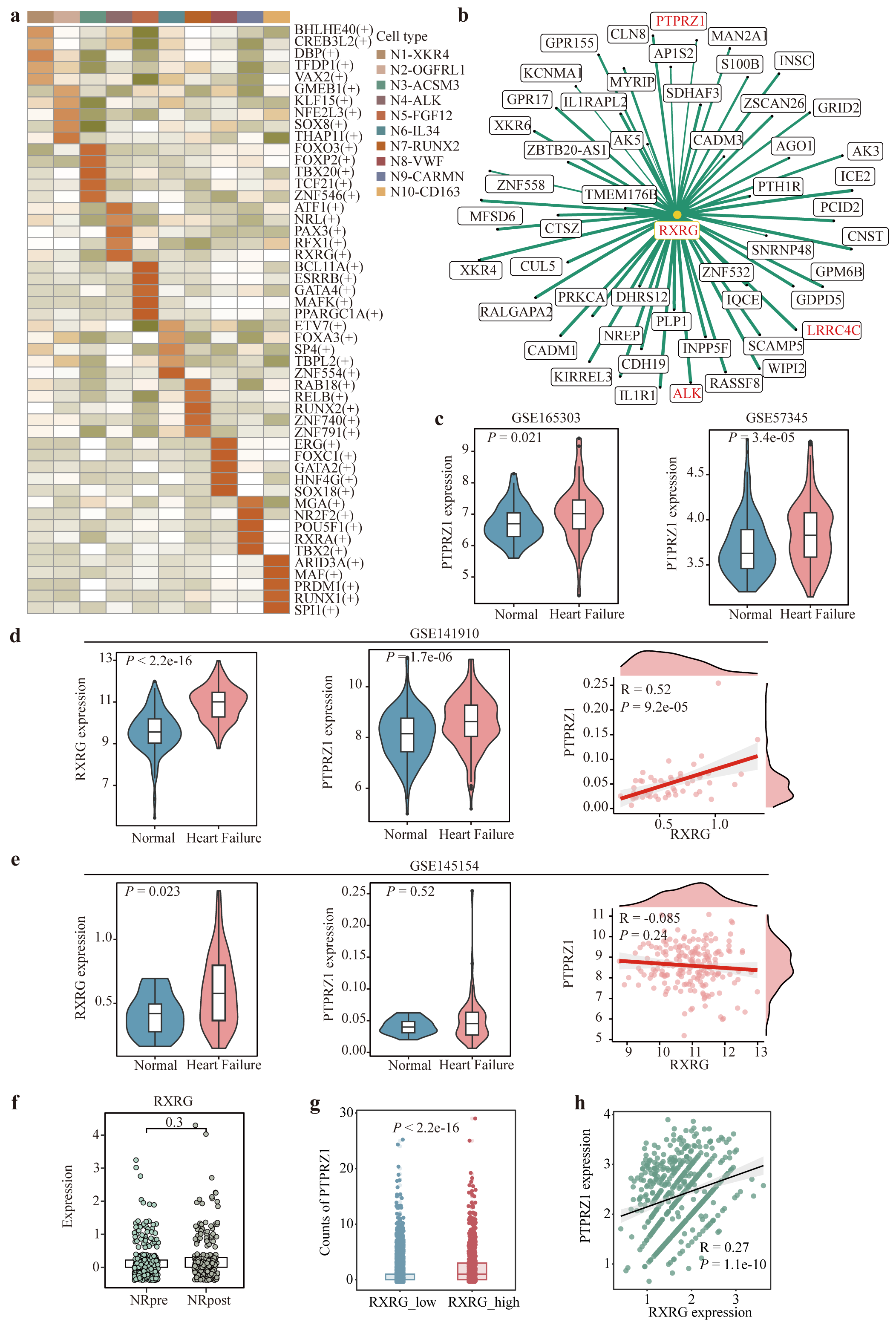

Supplement: S6 Fig — (a) The top five regulons with higher transcriptional activity in each neuronal subset. (b) The transcriptional network of RXRG with its top 50 targets. (c) Differential expression of PTPRZ1 in heart tissue between heart failure group and healthy group using GSE165303 and GSE57345, respectively. (d, e) Differential expression of RXRG (left) and PTPRZ1 (median) in heart tissue between heart failure group and healthy group in GSE141910 (d) and GSE145154 (e), respectively. Right Pearson correlation between the expression of RXRG and PTPRZ1 in heart failure samples based on GSE141910 (d) and GSE145154 (e), respectively. (f) Differential expression of RXRG before and after receiving LVAD implantation in patients who did not benefit from implantation. (g) Differential expression counts of PTPRZ1 between the RXRG_low (n = 6,538, cells with a zero count of RXRG) and RXRG_high (n = 831, cells with non-zero count of RXRG) neuronal cells from heart failure patients. (h) Pearson correlation analysis between RXRG and PTPRZ1 expressions in heart failure-neuronal cells with non-zero counts values (n = 565). In (c), (d), (e), (f), and (g), except for right plots of (d) and (e), Wilcoxon rank-sum test was used. (TIF) [file pcbi.1014082.s008.tif]

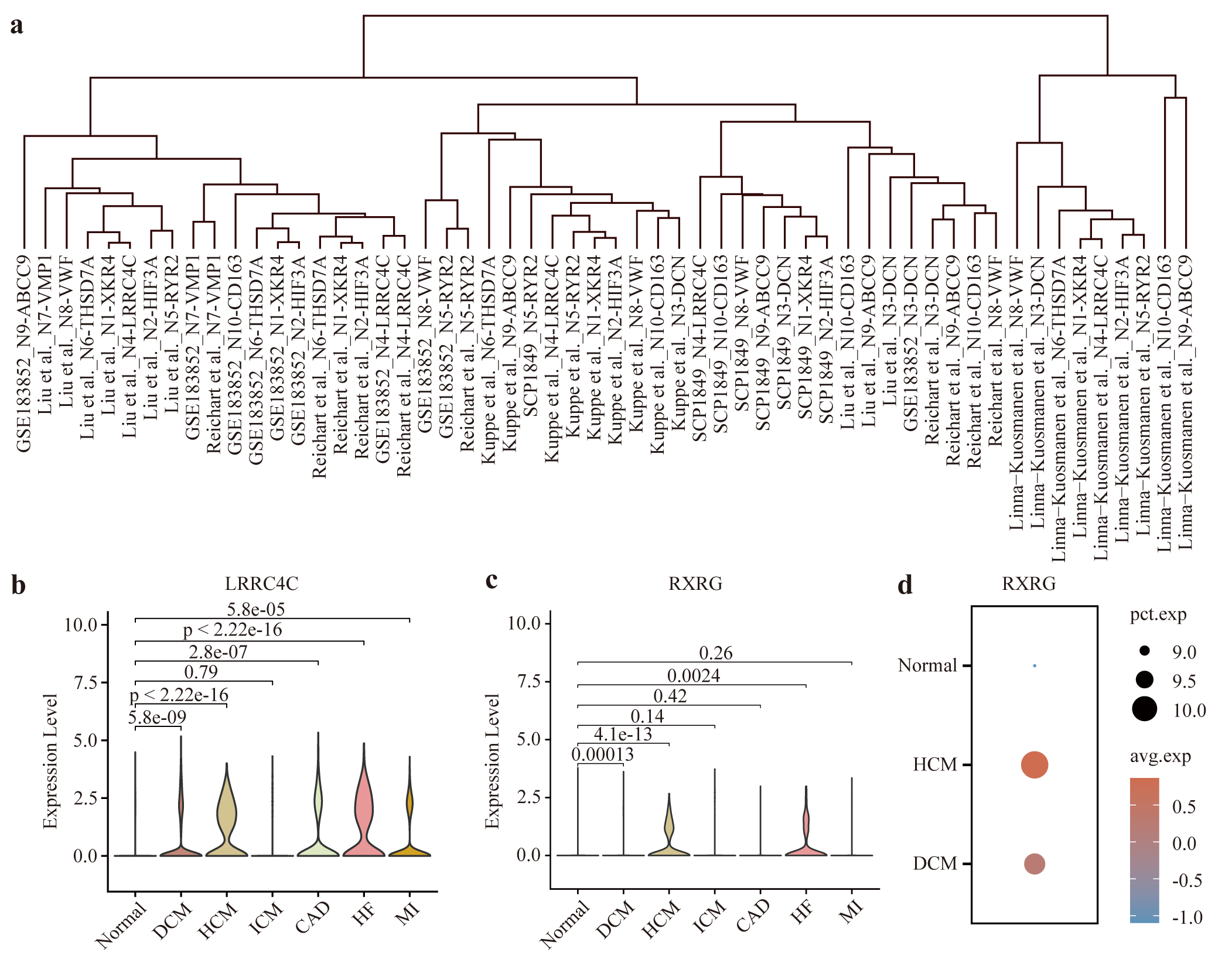

Supplement: S7 Fig — (a) Evaluation of similarity among neuronal subsets from different studies. (b, c) The differential expression of LRRC4C (b) and RXRG (c) between normal heart and diseased heart. Wilcoxon rank-sum test was used. (d) Distribution of the expression of RXRG in neuronal clusters from SCP1303. (TIF) [file pcbi.1014082.s009.tif]

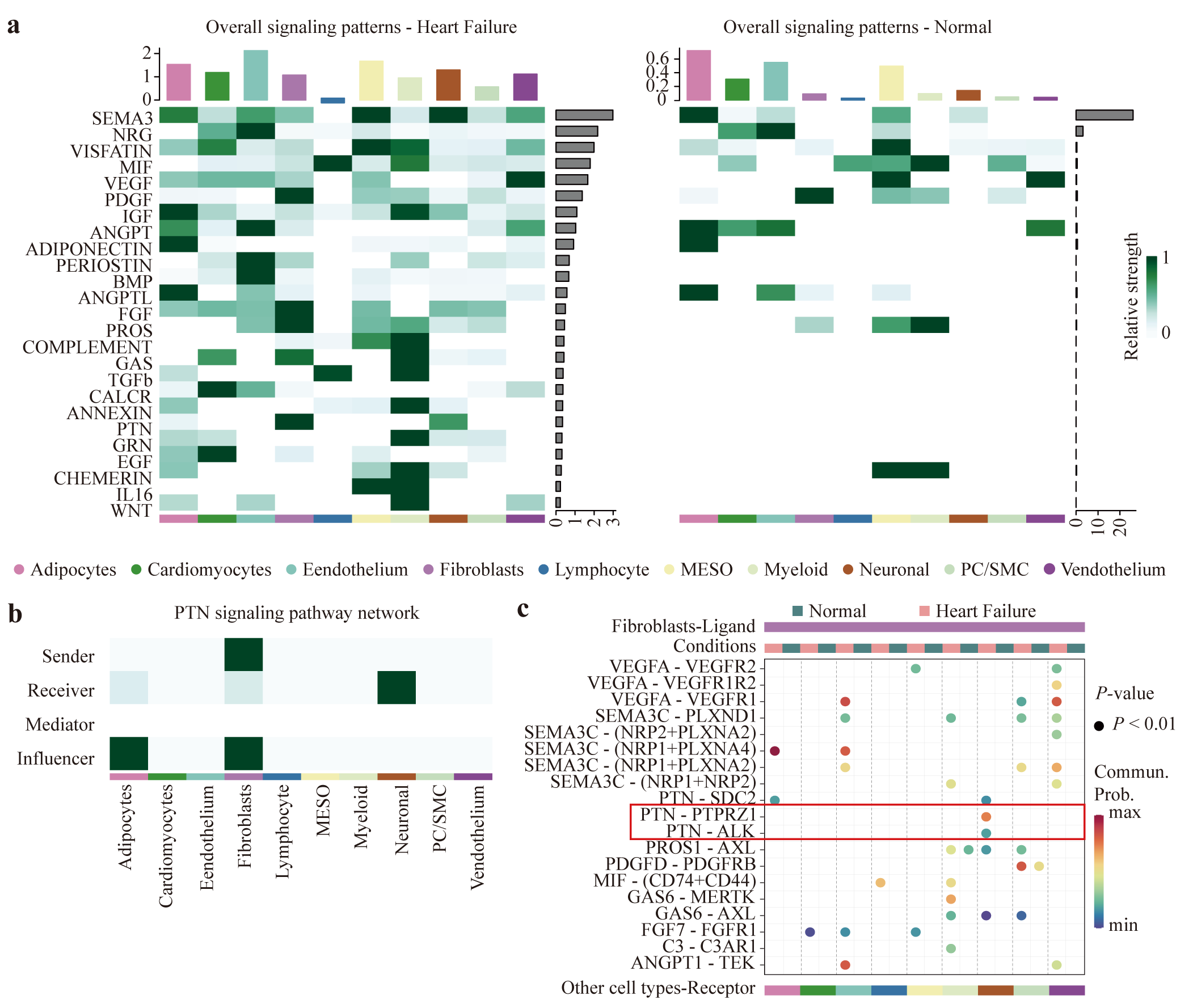

Supplement: S8 Fig — (a) The overall signaling of cell-cell communications in heart failure and healthy samples, respectively. (b) The communication strength involved in PTN signaling among cell types. (c) The communications between fibroblasts and other cell types in failing and healthy hearts. (TIF) [file pcbi.1014082.s010.tif]

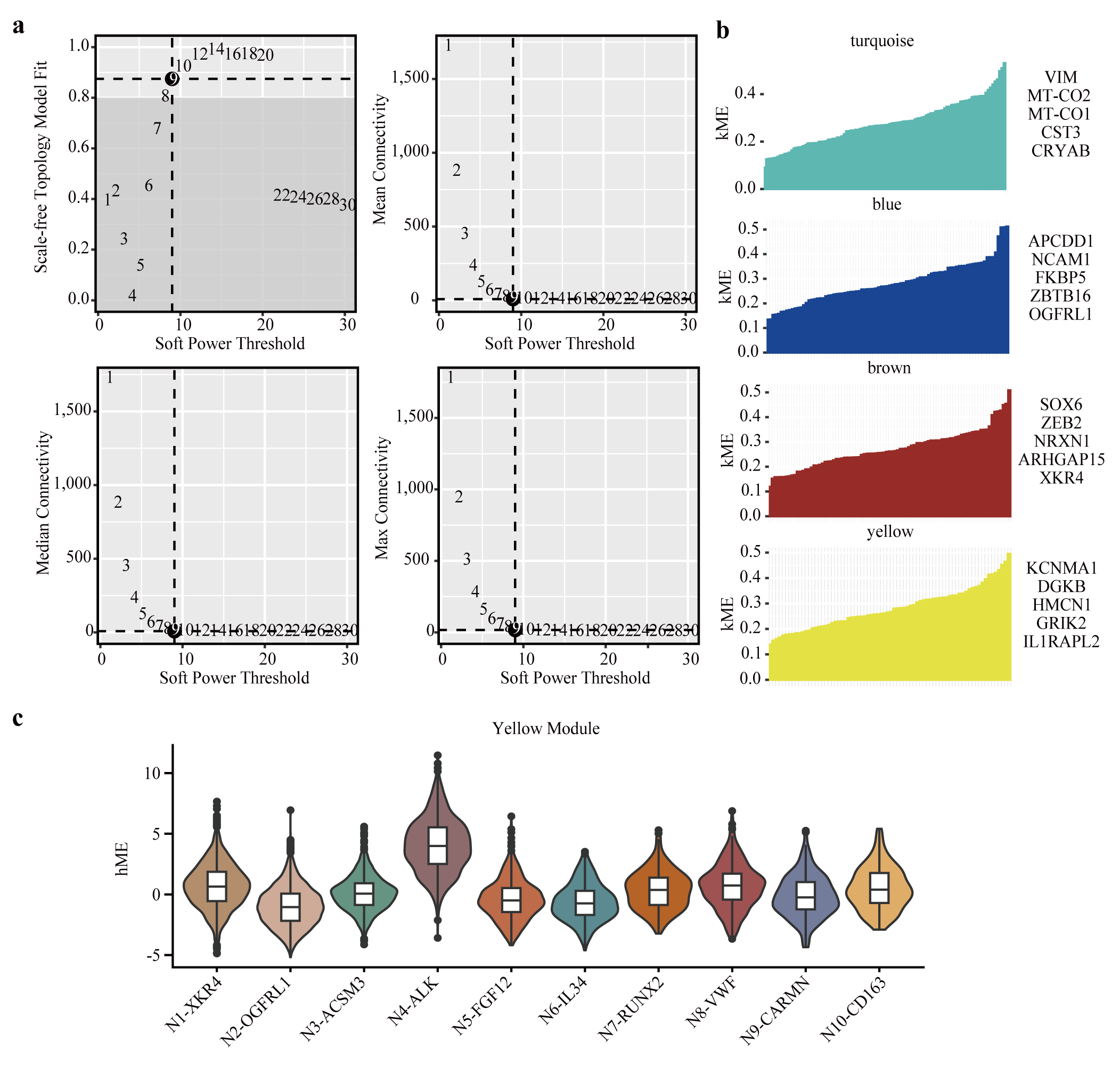

Supplement: S9 Fig — (a) Determination of the soft power threshold of the hdWGCNA algorithm. (b) Bar charts display the weights of hub genes in each module and listed the top five genes. (c) The average score of “yellow” module genes in the neuronal subsets. (TIF) [file pcbi.1014082.s011.tif]

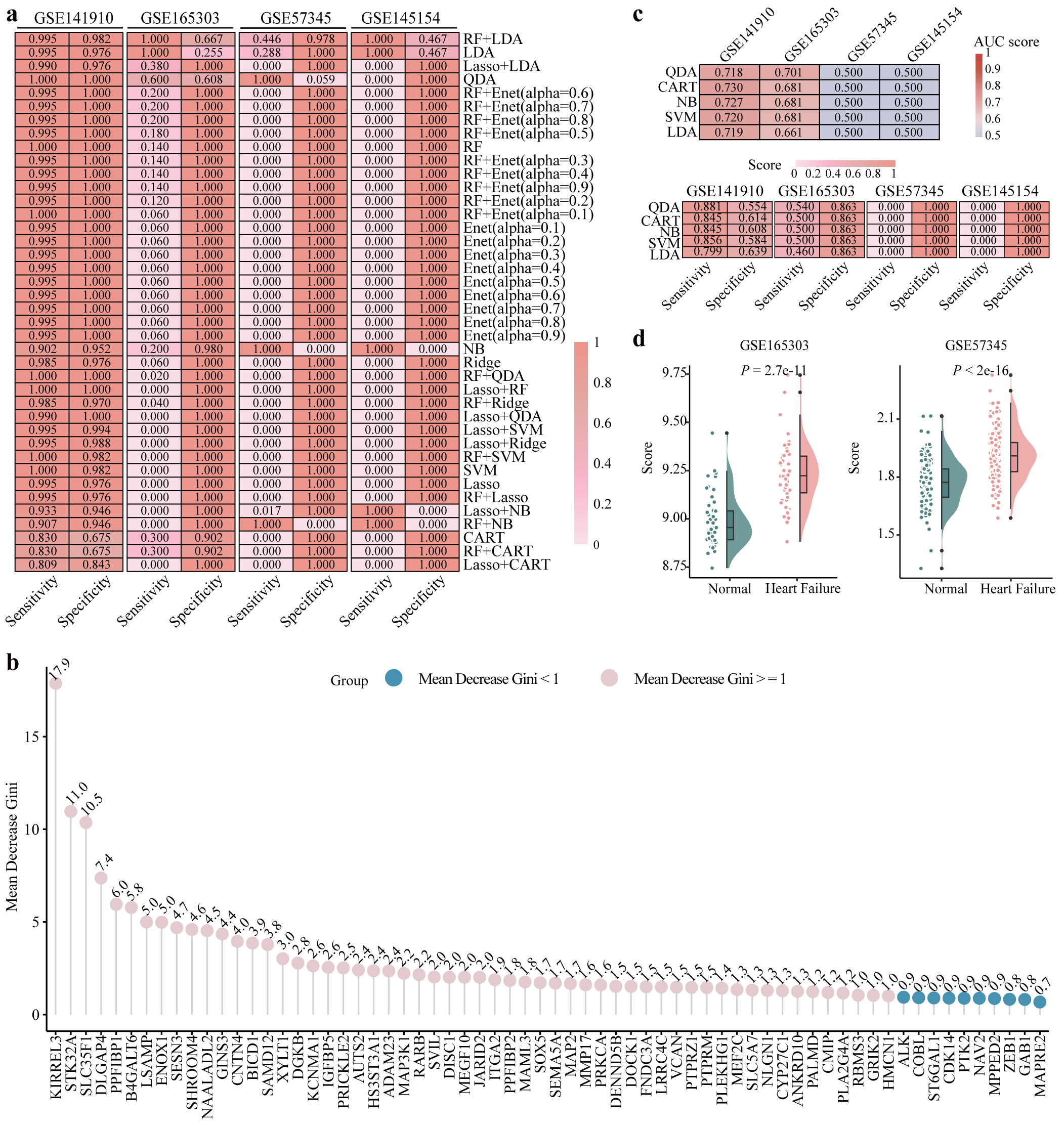

Supplement: S10 Fig — (a) Heat maps display the specificity and sensitivity scores of 40 heart failure predictive models across the four datasets. (b) Ranking of 66 identified genes based on importance scores from random forest feature selection, measured by the Mean Decrease Gini. (c) Evaluation of the predictive efficacy of NPPB expression for classifying heart failure patients. (d) The differential enrichment scores of 66 genes between the heart failure group and the healthy group in GSE165303 and GSE57345, respectively. Wilcoxon rank-sum test was used. (TIF) [file pcbi.1014082.s012.tif]
